# Developing and validating a holistic welfare assessment tool for zoo-housed great apes: Integrating resource-based measures with behavioural ecology insights

**DOI:** 10.1371/journal.pone.0340094

**Published:** 2026-01-30

**Authors:** Johanna Neufuss, Kirsten Pullen, Holly L. Farmer, Cerian Tatchley, Nick Davis, Jackie Chappell, Susannah K. S. Thorpe

**Affiliations:** 1 School of Biosciences, University of Birmingham, Birmingham, United Kingdom; 2 Chester Zoo, Upton-by-Chester, Chester, United Kingdom; 3 Wild Planet Trust, Paignton Zoo, Paignton, Devon, United Kingdom; 4 British and Irish Association of Zoos and Aquariums (BIAZA), London, United Kingdom; University Centre Sparsholt, UNITED KINGDOM OF GREAT BRITAIN AND NORTHERN IRELAND

## Abstract

Assessing the welfare of zoo-housed great apes is essential to ensure high standards of care, maintain zoos’ ethical and educational credibility, and support their conservation roles. However, welfare assessment remains challenging due to the complex cognitive, social, and behavioural needs of great apes and the limitations of existing evaluation methods. Many zoos rely on resource-based measures, which are easy to collect but lack the specificity needed to assess welfare accurately. In this study, we developed and tested a new standardized, evidence-based approach that integrated resource-based data with ecological and behavioural insights. This Ape Welfare Assessment, Resources and Ecology (AWARE) Tool was developed through collaboration with the Great Ape Welfare group (GAWg) and aimed to: (1) collect a holistic suite of resource and husbandry data that measures the constraints on the apes opportunities to express key naturalistic behaviours, (2) contextualize results within species-specific research on wild and captive great ape behavioural ecology and welfare, and (3) extend beyond existing zoo accreditation standards to support zoos aspirations for higher levels of welfare. Using data from zoo-housed chimpanzees, we tested whether this approach enhanced the validity of resource-based welfare assessments. Results demonstrate that the approach strengthens welfare evaluations and provides a more nuanced understanding of captive great ape wellbeing. We also propose a new validation framework for holistic welfare assessment tools such as this, where validating all individual indicators is neither feasible nor informative. Ultimately, we aim for the AWARE Tool to serve as a web-based resource for independent zoo use and formal welfare assessments. Future research will expand its application to gorillas and orangutans. This study represents a major leap towards establishing robust, species-informed and validated welfare assessment methodologies for zoo-housed great apes.

## Introduction

Measuring animal welfare is critical for modern zoological parks to provide empirical information on how zoo-housed animals are coping with their environment (e.g., [1] and see the World Association of Zoos and Aquariums [WAZA] Animal Welfare Strategy [2]). The welfare of captive great apes is of particular concern as they are long-lived and highly intelligent, inhabit large variable natural ranges, and are humans’ closest living relatives with whom we share complex cognitive, affective, and social abilities and needs. Meeting their behavioural, physical, and psychological welfare needs in captivity is thus challenging [e.g., 3]. However, failing to do so compromises the animals’ quality of life and the wellbeing of their caregivers [4], while diminishing their conservation potential by reducing their educational and research value. Moreover, zoos risk losing their social licence to operate if high welfare standards are not upheld. [5].

Animal welfare is a multidimensional concept comprising animal feelings, behaviour, health, cognition, resources and husbandry [e.g., 6–8]. In recent years, focus has shifted away from simply avoiding suffering or preventing harm, towards the provision of good welfare by providing animals with positive experiences, opportunities and conditions [9]. The Five Domains model focuses on four physical or functional domains: the animal’s environment, nutrition, behavioural interactions and overall health. The fifth domain is focused on the mental state, the animal’s feelings – positive and negative – that result from their experience of the previous four domains. While this model is central to WAZA’s Animal Welfare Strategy [2], and the animal welfare evaluations required of its members, the fifth domain is particularly difficult to quantify. Concepts such as agency (which refers to the ability of animals to engage in voluntary, self-driven, and goal-directed behaviour motivated by their own desires or needs) and competence (which results when an animal has the tools and strategies to deal with novel and ongoing challenges) are becoming increasingly central to the concept of positive animal welfare [10]. One example is the dynamic animal welfare concept (DAWCon [11]), which focuses on the dynamics of an animal’s environment over time, and the animal’s behaviour in response to changing environmental conditions. Coping with and adapting to these dynamics involves a continuous succession of positive and negative states. The authors argue that animals experience good welfare when they have the mental and physical capacity and opportunity to respond appropriately to the balance of positive and negative stimuli, events, and conditions over time [11]. Nevertheless, it’s important not to confuse resilience (which describes the ability of individuals to ‘bounce back’ from setbacks) with wellbeing in this model.

Two types of parameters are used for measuring the various aspects of animal welfare: resource-based (or input-based) measures are variables that reflect the physical environment and include the resources available to the animal and management practices, such as space allocation, housing facilities, bedding material, access to water, enrichment and food provision [12]. Animal-related (or output-based) measures include observing species-specific behaviour, appearance, and measuring health and physiological parameters [13]. These provide a more accurate reflection of an individual’s welfare state because they measure each individuals’ response to their physical and social environment and can indicate their emotional states [14]. Nevertheless, understanding the behavioural ecology of captive animals needs a multi-factorial approach since studies that only use single animal-based measures (such as corticosteroids; see review in [8]) are unlikely to capture the detail needed to obtain a holistic picture of an animal’s wellbeing [14–16].

Zoos commonly rely on resource-based welfare assessments because these are easy to assess quantitatively, are quick to collect and allow the welfare of many animals to be assessed rapidly, albeit at a fairly generic level. In addition, they do not require invasive methods, and the approach removes the need for complex analysis/interpretation. Resource-based perspectives also play an important role in the implementation of existing legislation, such as inspections related to the UK’s Secretary of State’s Standards of Modern Zoo Practice [17], and national or international zoo industry accreditation schemes and Best Practice Guidelines from the British & Irish [BIAZA], European [EAZA] and American [AZA] Association of Zoos and Aquariums. Nevertheless, evidence on which resource-based measures are important and how to promote good zoo welfare through this approach is still debated for many species [18], including all the great apes.

Moreover, there is a significant lack of validated and/or standardised welfare assessment tools [7,8]. This carries substantial methodological, ethical, and policy risks. When reliability and construct validity are not empirically established, welfare tools may either fail to detect low welfare or produce false alarms, which in turn can lead to misdirected interventions and inefficient use of resources. Such deficits can erode scientific credibility, reduce stakeholder confidence in welfare claims, and weaken the integrity of certification or assurance schemes. At the regulatory level, reliance on unvalidated measures can undermine the enforceability of policy and hamper progress toward meaningful welfare improvements.).

In 2018 two authors of this paper (SKT and KP who was then CEO of BIAZA) co-founded the Great Ape Welfare group (GAWg) in the UK. The aim of the group was to bring together researchers of wild and captive great apes, zoo practitioners and representatives of great ape-related welfare, regulatory and Government organisations to define best practice in great ape welfare, celebrate current successes, and to work together to generate tangible evidence-based improvements in welfare, husbandry and policy inter-linked with conservation and research. At the time of this study there were 14 members of the group. The aim of this project was to leverage GAWg’s expertise to create, test and validate a standardized, evidence-based great Ape Welfare Assessment, Resources and Ecology Tool (AWARE Tool) that provides an effective, yet rapid, assessment of zoo-housed great ape welfare by:

1) Integrating a holistic suite of resource and husbandry data to optimise information gathered by the tool. This ensures the tool incorporates all measures needed to ensure that welfare provision meets the mental and physical welfare indicators described above.
2) Applying the research literature on wild and captive great ape behavioural ecology and welfare to create the suite of indicators used in the tool and aid interpretation of the results. This ensures the tool assesses the extent to which the animals’ physical and social environment allows expression of complex, naturalistic behaviour. It also provides end-user readers with much-needed access to the underpinning research evidence in a single location and highlights where important gaps in knowledge and the evidence base exist.
3) Including components that go beyond the level of welfare required by the zoo inspection process, zoo-based accreditation and Best Practise Guidelines, so that each zoo will ultimately be able to use the tool to achieve the levels of welfare they aspire to.
4) Ensuring the tool can be applied robustly for multiple great ape species, in different settings and by different users.

In this first paper we use the chimpanzee (*Pan troglodytes spp*.) dataset to: a) test whether the AWARE Tool does strengthen a traditional resource-based approach, b) identify where refinements are needed and c) develop a validation framework for the tool. We do not provide an evaluation of the welfare status of zoo-housed chimpanzees as this is beyond the scope of the paper. Ultimately, we aim that the tool will be a web-based interface where zoos can upload their data for automated analysis and interpretation, as well as a tool that can be used for welfare assessment by relevant agencies. The results for gorillas and orangutans will be presented in future publications.

## Materials and methods

### Survey development

The survey underpinning the AWARE tool (S1 Survey) was created in 2019 based on GAWg expertise and extensive literature reviews on the behavioural ecology and welfare of each great ape species in the wild and in captivity. An updated and consolidated version of the chimpanzee literature review is included in the combined Results and Discussion section of this paper to contextualise both the selection and interpretation of the measures included in the survey. Incorporating GAWg’s diverse spectrum of experience allowed a wide range of perspectives on welfare to be reflected in the final selection. We created a single survey that could be applied to chimpanzees, gorillas, orangutans and bonobos, but was sufficiently detailed to reflect the specific needs of each species, when combined with the literature reviews. The questions focused on the individuals present; enclosure sizes, access, furniture, and frequency of modifications; environmental parameters; feeding, social groupings; welfare assessment and provision; acquisition, transfer and transition policies; enrichment planning and provision; capacity for the apes to exert control over aspects of their lives; positive reinforcement training; healthcare, and staff training and support. It combined quantitative questions with open text questions so zoos could expand on details or explain answers. The study was approved by the Ethics Committee of the University of Birmingham (ERN_25_4337). Written, informed consent was obtained from all participating zoos.

The survey was completed in two stages. In Stage 1, participating zoos completed a questionnaire independently. Stage 2 included questions requiring consistent measurements and categorizations (e.g., types of enclosure furniture and their orientations), so one team member (JN) visited each zoo to complete this section alongside the zoo staff. Zoo staff were not asked for personal opinions. Following data collection, each zoo was assigned a random number that was used in analysis and all data were treated anonymously. Where responses to specific questions would make identification of the zoo apparent, we have only presented generalised analysis. The survey was undertaken at nine chimpanzee collections in the UK and Ireland, but two zoos withdrew from the study.

### Survey analysis

Although ages of individuals are mostly well known in captivity, robust data on development stages are missing in the literature for captive populations [19]. We therefore defined the different age categories according to wild data [20–24]. These are: elderly adults (~33 + years), adult females (15 + years), adult males (15 + years), immatures (5–15 years), and infants (<5 years).

To reflect whether zoos were providing locomotor opportunities throughout all parts of the enclosures (particularly the highest levels) we classified enclosure height as low: ≤ 1 m above ground; medium: space between low-high) and high: ≤ 1 m below the ceiling (even if this area did not have locomotor supports), or the highest point of the outdoor climbing frame [after 16].

The enclosure features that particularly influence chimpanzee welfare and diverse behavioural expression are those that elicit natural physical and mental opportunities and challenges, such as the density and variety of arboreal supports and their flexibility, connectedness and unpredictability ( [16,25] and see Results and Discussion). To encapsulate these concepts in a simple but meaningful way we created Enclosure Complexity Indices (ECIs) for five parameters that translated the concepts of support complexity, diversity and temporal change from natural to captive habitats, through quantification of the number, structural variation, arrangement and temporal change of weight bearing supports. ECIs were calculated for indoor, outdoor and off show areas separately. They were scored numerically using thresholds or ratios to indicate simple, moderate and complex enclosure features. While this approach includes some subjectivity, the thresholds were based on logical boundaries that would be easy for users of the AWARE Tool to interpret and meaningful when compared to wild habitats. The parameters were:

1)
**Availability of flexible supports**: this was scored according to whether flexible supports were less, equal or more available than rigid supports. Flexible supports were defined as those that deflected underneath the animals’ weight. Equal availability was classed as the smaller number being within 20% of the higher number. Rigid supports included poles, logs, platforms, metal bars and steel mesh. Flexible supports included single ropes, cargo nets, hammocks, webbing and other objects including items that could be used for weight bearing such as barrels, tyres, etc.
2)
**No. of available supports relative to enclosure volume** (support density) to indicate the extent to which the zoos were utilising the available 3D space in the different enclosure types to provide species-typical locomotor environments such that the chimpanzees can move arboreally for sustained periods without descending to the ground. Maximum volumetric space was estimated by multiplying enclosure area (m^2^) by the maximum height attainable (m). This is a proxy for the true volume of the enclosure as attainable height for indoor enclosures without a roof mesh or climbing frame near to ceiling height will be lower than the actual height, and attainable height in outdoors enclosures will be dictated by climbing frames. Volume calculations for outdoor spaces may also overestimate usable volume because of containment barriers, since, for example, walls need jump distances kept clear. Nevertheless, this measure provides a broad indication of the complexity of the locomotor spaces. The output variable represents the average volume (m^3^) per support for each type of enclosure. Since the density of supports in chimpanzee habitats will vary substantially within and between wild habitat types, we adopted a biomechanical approach to classify thresholds based on chimpanzees’ long armspan and ability to cross gaps via bridging and jumping. We considered that indoor enclosures should be sufficiently dense to allow extensive arboreal movement, and thus selected an average of ≥1 support every 3 m^3^ as high density (as a rough indication that a large proportion of the enclosure allows arboreal movement) and ≤1 support every 10 m^3^ as low (indicating very limited ability to move arboreally), with the intermediate densities scored as medium. We considered that off-show enclosures should be slightly more densely filled because they tend to be smaller spaces but still need to offer choice and variety; high density was therefore an average of ≥1 support every 1.5 m^3^, low was ≤ 1 support every 3 m^3^ and intermediate densities were medium. Outdoor enclosures are often the largest, offer access to grassy areas as well and are much less likely to have roofed areas so maximum volume can rarely be used. For this high density was an average of ≥1 support every 10 m^3^, low was 1 ≤ support every 30 m^3^ and intermediate densities were again medium.
3)
**Extent to which zoos regularly changed supports in the enclosure**: according to whether they changed them only when things broke, 1–3 times per year, or more than 4 times per year.
4)
**Diversity of support types**: For the support types listed above we quantified diversity according to whether all the supports in an enclosure were of the same type; included 2–4 different support types or whether there were more than 4 different types of weight bearing support for each category of rigid, flexible and other supports.
5)
**Diversity of support orientations** Support orientations were classed as horizontal (± 20° from true horizontal), vertical (± 20° from true vertical) or angled (all other orientations). We reflected diversity according to whether all the flexible, rigid and other supports had the same orientation, mostly (e.g., more than approximately 50%) 2 orientations, or if all three orientations were equally represented in each support type.

### Statistical analysis

The sample (n = 7 responses) was too small to study using statistical analysis. Thus, qualitative analytical techniques were conducted using SPSS (v29), where these added clarity to interpretation of the data. Results are presented as values out of 7 unless otherwise stated. Twelve percent of questions were not relevant for some zoos and one zoo was missing data meaning it was not possible to quantify certain measures in the second part of the survey (such as off show area). In most of the Tables and Figures we have applied colour coding as a broad indicator of the level of welfare of each component, based on the evidence presented from the literature review. The aim is that this will support rapid visualisation of the results in the final AWARE tool for end users; highlighting where welfare is high and should be celebrated, and where small improvements/ modifications can make a positive impact on overall welfare.

## Results and discussion

### Overview of data collected with the AWARE Tool

**Fig 1:**
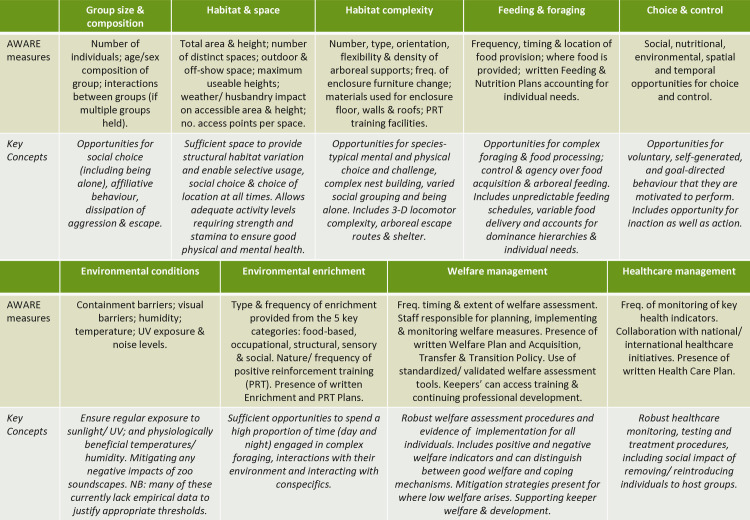
Overview of the measures included in the AWARE Tool survey and the welfare concepts they relate to.

Fig 1 summarises the resource and husbandry measures incorporated into the AWARE tool along with the key welfare concepts they relate to (and see S1 Survey for the survey questions). The measures fall into 9 categories: those that relate to the chimpanzees’ behavioural ecology (group size and composition, habitat and space, habitat complexity, feeding and foraging, and choice and control) are presented in the manuscript. The results for environmental conditions, environmental enrichment, welfare management, and health care management are presented in the Supplementary Results (S2 File) as these related more closely to logistical considerations. Our analyses combine the variation found in zoo provision with the relevant literature to test whether the chosen measures capture meaningful differences in welfare and to identify important gaps in the evidence base for captive care. We then assess the tool’s effectiveness and develop a framework for its validation.

### Group size and composition

Wild chimpanzees mostly live in large social communities with 20 to more than 100 members, including multiple females and their offspring, and multiple males [21,26]. These communities tend to be intolerant of non-group members and intergroup conflicts can be lethal [21,27]. However, with a fission–fusion social structure, they associate and travel most of their time in much smaller parties. Parties are characterized by a fluid membership that can last from minutes to several days and varies between populations. Overall, average party size is 6 individuals [28], but party size greatly increases when food availability increases, for example in the Kibale National Park in Western Uganda (*Pan troglodytes schweinfurthii* inhabiting a moist tropical forest that supports a high density of primates), average party size is 10 but can increase to 47 individuals during periods of high food availability [26]. Party size also increases when estrous females are present [26]. Such social fluidity gives individuals considerable freedom to choose whom to associate with and to spend time alone.

While social behaviours only form a small part of the daily activity of wild chimpanzees (e.g., 4% of a 24-hour period [29]), social deprivation is considered to be one of the main causes of undesirable behaviours in captive individuals [30]. Some authors have suggested that captive chimpanzee groups should mimic the size of traveling parties because large, static groups are not the normal social unit for wild chimpanzees [29]. Other studies suggest that groups of seven or more chimpanzees perform increased affiliative behaviour compared to smaller groups [28,31] and that pairs, trios, and even small groups of 4–6 individuals may not provide the social complexity required to meet the social needs of captive chimpanzees [32].

The survey obtained demographic data on the individual chimpanzee and on interactions where multiple groups were held groups (S1 Survey). However, all seven zoos in this study held a single chimpanzee group. Overall group sizes ranged from 3–21 individuals with a mean size of 9.57 (SD: 6.24) chimpanzees. While two zoos held more than 15 individuals, three held less than 6 individuals. At face value, only the smaller groups sizes would stand out as indicating groups where the chimpanzees’ may lack opportunities to make meaningful decisions about their social interactions. However, a group can be of supposedly optimal size yet meet few of the social, reproductive or psychological needs of an individual if the structure and composition are not appropriate. In wild chimpanzee communities’ females outnumber males [33] and immature and infant chimpanzees represent a large proportion of the group, e.g., 34% and 17% respectively for M group in the Mahale Mountains National Park in Tanzania (*Pan troglodytes schweinfurthii* inhabiting a mosaic woodland habitat with pronounced wet and dry seasons) [34]. All groups in this study had more adult females than adult males (overall total 39% male, 61% female). Individuals ranged in age from 2 months to 53 years old, and the means for each zoo ranged from 20.50 years (SD: 8.36) to 37.33 years (SD: 10.12). However, just 8% of the sample was classified as immatures or infants since most of the zoos were either not breeding at the time of the study or had no breeding group. Sixty one percent were adult and 31% were elderly adults.

The impact of a skewed group composition can be seen in social stability. Play is one example of a potential output-based indicator of good welfare in captivity because it often disappears when animals are under survival threat; is thought to be accompanied by pleasurable emotions, and is socially contagious and thus can spread a good welfare state in groups [5,35]. There is some evidence from the wild that young individuals have a positive welfare effect on the social dynamics of chimpanzee groups, such as encouraging older individuals to play. For example, Matsusaka [36] found that only 12 out of 793 bouts of social play occurred between adults, whereas the remaining 781 bouts included immature individuals. Whether the presence of more playful, younger and older individuals cause welfare improvements in captive chimpanzee groups has not yet been confirmed empirically. One study found no evidence that the presence of youngsters in a group reduced abnormal behaviours [37], while other studies show that groups that contain a range of age/sex classes possess higher social complexity [29,38]. Play is also critical for the individuals’ development. It is a key mechanism by which youngsters can build competence and musculo-skeletal strength, and exercise agency through choice and control [9].

Wild chimpanzees also display high levels of intra-group aggression: Hunt [39] estimated that a wild chimpanzee can expect to be the recipient of aggression/ attack once every 44 hours. As a result, captive management must balance the needs of chimpanzees to have companionship with other chimpanzees with the need to minimize wounding resulting from natural intraspecific aggression. Some research suggests levels of aggression can be higher in captivity than in the wild [40–42], and in some cases such aggression can be fatal (although this is also seen in wild populations [43]. However, aggression is also influenced by the demographics and personalities of the individuals involved, which likely explains Seres and colleagues [44] showing that large groups can be formed with a minimum of wounding and a group of up to 29 individuals that were successfully managed with relatively low aggression at Chester Zoo [45]. Nevertheless, current evidence suggests that mixed-age and mixed-sex groups are the most socially stable and have the least risk of wounding [42,46].

Overall, evidence from the literature and the sampled collections indicates that incorporating assessments of individuals’ capacity for social choice, including options to escape and be alone, is essential for identifying situations in which welfare potential may be constrained, even when resource-based indicators alone suggest satisfactory conditions (Fig 1). Enhancing a resource-based assessment to yield a more refined picture of the social opportunities and constraints experienced by individuals, necessitates integrating this information with environmental variables such as enclosure area and height, number of rooms, and habitat complexity to determine whether potential constraints arising from group composition are mitigated or exacerbated by the physical environment. Thorpe and colleagues [25], for example, reported a reduction in attack rates from 0.32 to 0.07 per hour of observation among sanctuary-housed chimpanzees when small enclosures were modified to enhance physical complexity and reduce stress.

### Habitat and space

Wild chimpanzees inhabit a range of habitats, including tropical and montane rainforests, swamp forests, forest-savannah mosaics, and even dry savannah (e.g., [47]). They can choose where, when and how far to travel, although predation risks and social, territorial and foraging needs clearly constrain movement patterns. The size of their home ranges vary between 7 km^2^ and 60 km^2^ [21,48–50]. Overall daily travel distances also vary, with most populations tending to travel 2–5 km (e.g., [24]), but some travelling up to 10 km per day [51]. Translating wild home range sizes and daily travel distances into recommendations for how much space is needed in captive settings is challenging because wild animal maximum range sizes and travel patterns are strongly influenced by the group’s need to feed, in environments where resources can be widely distributed and fluctuate in availability. Sex and position in the dominance hierarchy also influence space use for chimpanzees: males often range over the entire territory and use an area that is 1.5 to 2 times greater than that of females [52]. Wild females have core areas, with the highest-ranking females dominating the areas with the greatest abundance of preferred foods. The core areas of high-ranking females are also smaller, affording them higher feeding efficiency through lower travel costs and intimate knowledge of resources [53]. Space then, independent of its complexity, is a key mechanism by which wild chimpanzees organise themselves socially, and reduce tension and resource-related aggression. For that reason, overall space should be considered independently in welfare assessments, in addition to as part of the overall complexity of the available habitat (Fig 1).

Larger zoo enclosures have been shown to encourage significantly greater behavioural diversity than smaller spaces; including more foraging, travelling and patrolling [3,54,55]. Chimpanzees that were relocated from a small indoor facility (27.87 m^2^) to a larger indoor and outdoor enclosure (587 m^2^) nearly doubled their time spent travelling (from 4.94% to 8.82%, [56]). Like wild chimpanzees, those in captive settings also seem to use only a small fraction of their available space at any given time [54]. Chimpanzees at Lincoln Park Zoo for example spent half of their time in only 3.2% of their available space [57]. Ross and Schendar [54] studied daily travel distances of adult chimpanzees for 5 years when they were restricted to their indoor space (20.75m^2^ per chimp) and when they had access to their outdoor enclosure, which provided nearly 6 times more floor space per chimpanzee (122.95m^2^). They found that daily travel distances averaged 1.18m/min indoors compared to 2.32m/min when given outdoor access as well. This means that 6 times more horizontal area only led to 2 times more daily travel. The chimpanzee daily travel distance averaged 1.39 km/day when given access to their full indoor–outdoor environments [54]. This still falls short of typical wild chimpanzee travel distances (2–4 km/day), especially since the captive values included vertical distances, whereas wild values are only horizontal travel distances, and some wild chimpanzees can spend 50% of their day in the trees [58]. Overall, activity levels (which are strongly influenced by travel distances) are tightly associated with health: heart-related issues are the leading cause of death for captive apes [54,59,60] and heart health in humans is closely correlated with obesity and exercise [e.g., 61]. Degenerative joint conditions are also stimulated by low exercise in humans (see [62] for review). Of course, activity levels can also be increased by increasing enclosure complexity, height and enrichment, but the key points here are 1) that measuring available space in an enclosure does not directly reflect the space the chimpanzees will access, and 2) when considering modifying enclosure sizes or building new enclosures, increasing *space used* by a desired amount will require increasing *total available space* by a much greater value. This scaling effect could explain why some authors have concluded that chimpanzees only receive moderate benefit from increases in enclosure space [63].

Overt use of space is not the only relevant consideration in determining the effect of space on ape welfare, given that the perception of space and the choice to access additional space can also confer behavioural benefits, even when that space is not used [57]. A key challenge in the calculation of space requirements is that we don’t know how chimpanzees perceive the space around them and how this is influenced by factors such as enclosure furniture, physical/visual barriers and the social dynamic of the group. The presence of female core areas in the wild according to dominance [53], and variation in daily travel strongly indicates that there will be spatial segregation in zoo chimpanzee habitats so that not all individuals can access all areas of the enclosure. Larger spaces therefore maximise the chance that even low ranking individuals can access sufficient space to exhibit natural behaviours, escape from attack, make meaningful choices about where they want to be and who they do and do not want to be near to, gain sufficient exercise to maintain healthy musculo-skeletal systems and experience sufficient variability over space and time to be cognitively challenged and have control over their lives [e.g., 16, 56, 64–67] (Fig 1).

Other research indicates that the number of distinct areas that captive chimpanzees can access is also important for their welfare. Having the choice to access linked enclosure rooms or an outdoor area has been shown to positively affect behaviour, such as increasing positive social behaviours [68] and the expression of social preferences and fluid group membership [3]. Accessing outdoor areas is associated with decreased abnormal behaviour, higher activity levels and possibly more enrichment use [69], although this is also obviously linked to habitat complexity. The height of the enclosure is also extremely important. In the wild chimpanzees climb to substantial heights and many spend a large proportion of their day off the ground. When chimpanzees travel arboreally, they often move through the forest canopy at heights of over 30 m (e.g., [70]). In captivity vertical space also adds diversity, increases the usable space for a smaller ground footprint, and provides opportunities for natural social stratification [71] and the ability to distance themselves from human visitors. Ross and Lukas [72] showed in one group of zoo-housed chimpanzees that individuals used all vertical tiers of a 7.6 m high indoor space but showed preferences for the area closest to the ceiling. AZA recommends that an appropriate indoor/outdoor exhibit space should have minimum useable vertical heights of over 6.1 m and at least 4.6 m in off-show areas. Whether these vertical height recommendations are sufficient to optimise welfare remains unclear however, as no studies have yet quantified the link between wellbeing and variation in accessible height. Vertical heights in captivity are clearly much lower than the tree heights that chimpanzees routinely climb in the wild and thus it is reasonable to suggest that greater heights can improve welfare for the reasons cited above. Yet, we need more robust evidence to guide minimum vertical heights for chimpanzee enclosures.

In this study all zoos provided multiple distinct spaces available for the chimpanzees (ranging from three to nine indoor, outdoor and off-show areas). Three had multiple indoor enclosures and six provided multiple off-show areas. One zoo didn’t have an on-show indoor space and one didn’t have an off-show space. While all indoor enclosures had multiple access points to prevent dominant chimpanzees monopolising doorways, three only had one access point to outdoor enclosures. Most off-show spaces also had multiple access points (Table 1). Overall, the total space available (indoor, outdoor and off-show) ranged from 525m^2^ - 2940 m^2^; both extremes of this range were in zoos that housed ≤ 6 chimpanzees. The amount of space in relation to the number of chimpanzees varied substantially between zoos: indoor space ranged from 5m^2^ - 60m^2^ per chimpanzee; outdoor space ranged from 55m^2^ – 917m^2^ per chimp, off-show space was mostly 3m^2^ - 5m^2^ per chimpanzee (except for the zoo that didn’t have an indoor on-show enclosure, where the off-show space was considerably larger than the other zoos). The total area available per chimpanzee ranged from 88m^2^ – 980m^2^. The maximum useable height of the enclosures also varied: indoors ranged from 3.5m – 12m; outdoors from 6m - 12m and off-show from 2m - 6.2m. The zoo that had the smallest indoor area per chimpanzee and one of the smallest outdoor areas per chimpanzee, had one of the highest useable heights indoors and the highest useable height outdoors (12m). Questions on resources were augmented by questions on usage; when asked which age groups used the maximum height available on a daily basis, respondents reported that 100% of adults used the maximum indoor, outdoor and off-show heights; 67% of elderly individuals used the maximum indoor and off-show heights but only 29% of elderly individuals used the maximum outdoor height.

**Table 1:** Enclosure details for each group of chimpanzees, classed according to group size.

No. chimps per group	≤ 6			7-14		≥15		Mean (SD)
**No. of rooms/ spaces**	6	3	8	8	9	9	8	9.57 (6.24)
**Total available area (m**^**2**^)	2511	525	2940	1123^a^	992	1848	1996	1705.00 (867.9)
**Total available area per chimp (m**^**2**^)	419	88	980	141^a^	124	123	96	281.23 (328.85)
**Total indoor area per chimp (m**^**2**^)	11	33	n/a	60	9	22	5	23.06 (20.60)
**Maximum indoor height (m)**	4	3.5	n/a	8	4	12	12	7.25 (4.02)
**Total outdoor area per chimp (m**^**2**^)	405	55	917	81	111	98	86	250.27 (317.56)
**Maximum outdoor height (m)**	10	10	8	6	6	8	12	8.57 (2.23)
**Total off-show area per chimp (m**^**2**^)	3	n/a	63	? ^a^	5	3	5	15.66 (26.47)
**Maximum off-show height (m)**	2.5	n/a	6.2	4.0	2.0	2.4	3.3	3.40 (1.55)
**>1 access points to all Indoor. Outdoor. Off-show rooms?** ^**b**^	Y.N.Y	Y.Y.Y	N/A. N.M	Y.N.Y	Y.Y.M	Y.Y.Y	Y.N.M	

Indoor and outdoor areas are classed as publicly viewable areas. Off-show areas are the primary off-show areas that the apes regularly use and do not include secondary off-show areas such as those specifically used for medical treatment, quarantine etc.

^a^The total off-show area was not available during the survey visit (denoted by?), resulting in total area available not accounting for this space.

^b^Y: yes; N: no; M: most; N/A: not applicable;

Further questions about usage quantified whether the chimpanzees had constant, variable, or no access to each area across day and night and summer and winter (excluding short closures for cleaning or other husbandry activities) to augment the overall space data with the potential minimum space experienced by each individual (Table 2). Access to the indoor enclosures was the most frequent and consistent, access to the off-show areas was frequently blocked (or not available) and access to outdoor space was the most variable, largely because of the weather. Table 2 shows that variation in the actual space available per chimpanzee ranged from 88m^2^ - 917m^2^ during summer days and 25 m^2^ - 980m^2^ during summer nights. In the winter the range reduced to 5m^2^ - 980m^2^ during the day and 10m^2^ -980m^2^ during the night. The maximum reductions equated to >90% space loss (although to understand the relevance to welfare, each result must ultimately be viewed in relation to the original size). Even at zoos that remained open during bad weather, the chimpanzees would presumably choose not to use outdoor space except in extremis, although a substantial amount of this time will have been spent sleeping.

**Table 2:** Chimpanzees minimum access to each area, day and night, during the summer and winter.

Zoo		Indoor		Outdoor		Primary off-show		Min. area per chimp^a^ (m^2^)		Max. reduction in space per chimp^b^ (%)	
		**S**	**W**	**S**	**W**	**S**	**W**	**S**	**W**	**S**	**W**
**1**	Day							91	5-91	5	95
	night							96	10-96	0	90
**2**	Day							120	120-124	3	3
	night							116	116	6	6
**3**	Day							120	120	2	2
	night							25	25	80	80
**4**	Day				#			405	11-416	3	92
	night			#	#			35	14-35	92	92
**5**	Day				?			141	?	?	?
	night				?			141^c^	?	?	?
**6**	Day					n/a	n/a	88	88	0	0
	night					n/a	n/a	88	33-88	0	63
**8**	Day	n/a	n/a					63-917^d^	980	6	0
	night	n/a	n/a					980	63-980	94	94

S: summer, W: winter. Dark blue: no access; mid blue: access varies according to outdoor weather conditions and husbandry; light blue: full access (except for short closures due to cleaning), green: nighttime data.?: missing data/ insufficient information in survey response; n/a: does not have that type of space; #: access was provided to part of the outdoor enclosure.

^a^Where access varies according to the weather/ husbandry (mid blue), ranges for minimum space are provided to reflect the variation.

^b^The maximum reduction in space represents the worst-case scenario and was calculated as the difference between the theoretical maximum space available if all enclosures were accessible and the minimum actual space accessible during summer and winter when seasonal variations caused closures.

^c^Off-show area data was not available, so actual accessible values will be higher than those presented here.

^d^Z8 chimpanzees chose each summer morning if they wanted to be inside or outside, so the ranges reflect all chimpanzees staying in and all chimpanzees going outside.

Given the limited heights indoors and off-show, reductions in space due to husbandry management and poor weather could have profound implications for the chimpanzees’ ability to express natural behaviour. For example, chimpanzees reduce how often they actively seek interactions with others or even decrease all social interactions when proximity between individuals is forced by being restricted to an indoor enclosure [45,73]. Similarly, when chimpanzees are confined in small spaces it can lead to increased aggression or self-directed behaviour [57] as they have significantly decreased opportunities to make social adjustments to alleviate social tension. Chimpanzees often employ coping strategies in response to stress experienced during spatial restriction. Coping mechanisms to stress are, however, meant to be temporary states and can become a problem when they change from a temporary state into a long-term condition or trait, such as abnormal behaviours (see review in [74]). Space itself is a crucial resource as it provides a physical area in which to perform behaviour and regulate group social dynamics. When individuals have limits on their inter-individual spacing and when coping does not enable them to change into a positive state, they experience long-term stress and increased abnormal behaviours when enclosures are too small or access to larger areas is restricted [57,74,75].

Ross and colleagues [57] propose that there is likely to be a ceiling effect at which point the behavioural benefits of providing more space begin to level off. However, until we can distinguish between need and ability to cope; have identified the scaling effect and the benefits of additional space from all five of the 5 Domains Model, we must be cautious about identifying minimum or maximum enclosure sizes. The design of new enclosures, in particular, should seek to maximise both horizontal and vertical space. Overall, these results show that collecting nuanced data on the minimum accessible space when weather is poor or parts of enclosures are closed for other reasons, greatly enhances the power of the information available for understanding the welfare experienced by individual chimpanzees.

### Habitat complexity

Although wild chimpanzees are considered to be largely terrestrial, they can spend about 50% of their day on arboreal supports [76] and up to 18% of their total locomotion time off the ground [39]. The forest canopy creates a complex three-dimensional network of trunks, branches and vines that vary in diameter, orientation, flexibility, and connectedness and that change over time due to seasonal variation, growth and decay. Their broad repertoire of locomotion and posture allows them to control flexible branches, make nests, avoid falls, change height in the canopy, cross arboreal gaps and escape attacks [e.g., 24,72,77]. Of all the great apes, chimpanzees travel most on the ground and then climb vertically to access food and other arboreal resources and to escape from attacks [58]; 85% of their feeding behaviour is arboreal. Physically demanding climbing and descent behaviours and suspensory postures are required to facilitate arboreal feeding from flexible supports [39,76]. Up to 24% of their arboreal locomotion uses supports that are < 4 cm in diameter and are therefore highly flexible and up to 26% percent uses supports from 4–10 cm in diameter, most of which will deflect under their weight [58]. These flexible supports not only challenge the chimpanzees’ musculoskeletal system, but their properties alter dynamically as the individual moves on them, so they are also cognitively challenging. The forest also provides shelter during bad weather.

Encouraging arboreal activity in captive apes is essential for musculoskeletal health since it places large forces on the body in a wide range of joint positions, which helps to prevent degenerative joint diseases, such as arthritis. The ‘Enclosure Design Tool’ process has shown that employing a biomechanical approach that focuses on making enclosures *behave* like the natural environment rather than *look* like it, ensures captive chimpanzees experience the kinds of environmental challenges wild chimpanzees face on a daily basis [16,25]. A core component was a flexible 3D grid made from car seat belt webbing, which required the chimpanzees to adjust their locomotion and posture dynamically to adapt to the movement of the support under their body weight. The grid’s flexibility also changed according to how many chimpanzees were moving or sitting on it, creating a level of unpredictability, that was both physically and mentally challenging. Its attachment points and flexibility could also be easily changed over time. Combined with modifications to enrichment and husbandry this process increased the percentage of time spent off the ground from 8% to 54% and the percentage of off-ground time moving on energetically-demanding flexible supports from 1% to 64% [25].

Other studies have shown that, when given appropriate enclosure furniture, captive chimpanzees spend up to 75% of their time off the ground, similar to their wild counterparts (e.g., [56,71,78–80]. Such furniture includes climbable walls and ceilings, ledges, platforms, hanging hoses, tires, and cargo nets [56]. While mesh can be evocative of the small cages historically used for zoo animals and is expensive to use for large areas, it can greatly enhance the welfare of great apes because it: optimises the chimpanzees’ ability to utilise all of their 3D space; provides a strong base to attach flexible climbing frames too that can be easily changed over time and allows escape pathways for subordinate animals [25]. Studies have also demonstrated that captive chimpanzees often prefer the highest vertical space available [64]. Connected vertical structures (including ropes and hammocks) positioned throughout the full 3D space of the enclosure, combined with flexible visual barriers to create distinct spaces within each enclosure provide arboreal escape opportunities that allow chimpanzees to avoid physical and visual contact with each other, significantly reducing severe aggression and social stress [16,25,81]. Conversely a lack of 3D space and arboreal escape routes has been shown to increase aggressive behaviour in captive settings [38,81]. Accessible off-ground enclosure features are therefore particularly important for creating meaningful distance between dominant and low-ranking individuals during aggression, particularly in smaller enclosures.

Ross et al, [57] assessed the impact of both space and complexity in a holistic longitudinal study of chimpanzees and gorillas that were transferred to new facilities, which increased available space for each of the five chimpanzees by approximately 15m^2^ [63] (unfortunately it is not possible to calculate the percentage increase in space). They found that abnormal behaviours reduced by 73% and attention behaviours (indicative of being alert and vigilant) reduced by 82%. Both were statistically significant. Ross and colleagues [57] summarised the overall chimpanzee and gorilla results as ‘conservative’ and this phrase was repeated by Angley [63] for chimpanzees, but the results need to be viewed in context. The change was not from small to large or from simple to complex, but from a 7 m high, functional, indoor, hardscape setting to a 10 m high modern, indoor-outdoor, naturalistic setting with a deep mulch floor, and some meshed walls to increase climbing surfaces [57]. The magnitude of the reduction in abnormal and attention behaviours is therefore striking. These results were accompanied by (among others) a reduction of 27% in self-directed behaviours and 87% in agonistic behaviours and an increase of 15% for feeding/foraging. Although not statistically significant, these results combine to show that a substantial impact on the holistic welfare of the chimpanzees accrued from a transfer to a slightly better enclosure. The study also found a 22% increase in inactivity. Taken alone this could be viewed negatively but it seems to be explained better by the reduction in self-directed behaviours and vigilance/attention, plus an increase of 30% in subjects touching or being within 1 m of a conspecific, indicating a socially calmer grouping in which chimpanzees *can* rest in frequencies that are more parallel to wild data [57,82].

The chimpanzee enclosures in this study contained a variety of support types, such as rigid (poles, branches, logs, tree trunks, climbing holds, climbing frame, nest baskets, platforms, mesh ceiling, rocks, stones); flexible (ropes, webbing, cargo nets, firehose hammocks, firehose straps, browse); and other objects (tyres, plastic balls, barrels, platform feeders, hanging feed-boxes). All zoos provided outdoor climbing frames and most provided off-ground climbing furniture at all three height levels in their indoor exhibits. Much greater variation was found however in the provision of off-ground locomotor supports at all three height levels in off-show areas; in general, the highest levels had the fewest locomotor supports.

**Fig 2:**
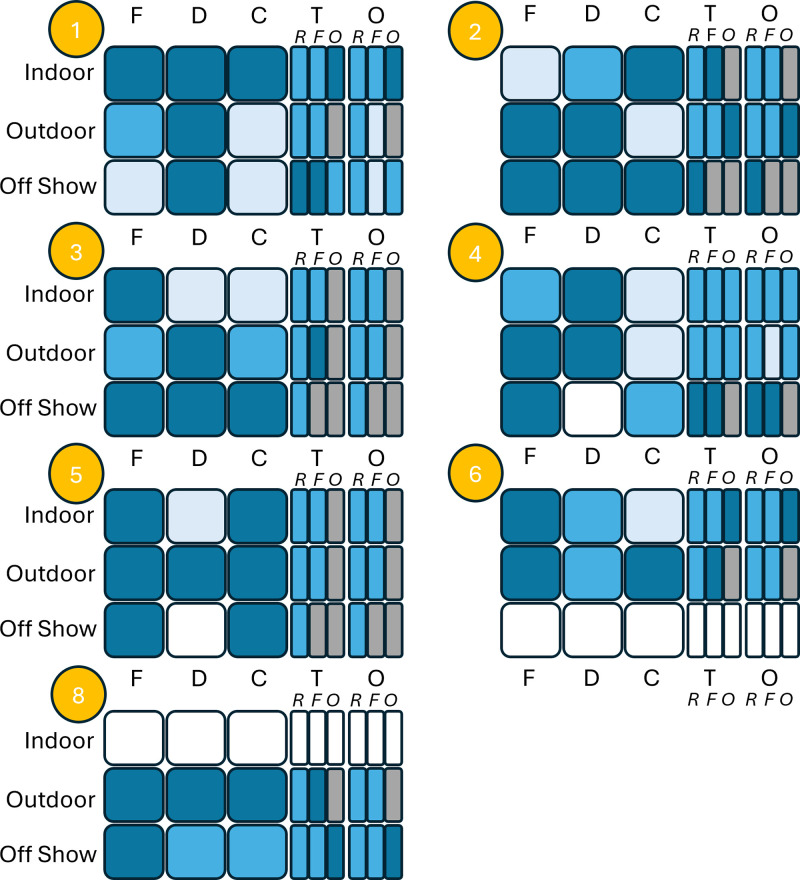
Enclosure Complexity Index grid. Zoo codes are in orange circles. F: proportion of flexible vs rigid supports; D: density of supports; C: extent to which supports are changed over time; T: diversity of support types, O: diversity of support orientations. For type and orientation, the letters in italics indicate supports that are rigid **(R)**, flexible (F) or other **(O)**. Dark blue cells indicate the parameter was scored as low complexity; mid blue indicates moderate and pale blue indicates high complexity. White cells indicate that the zoo did not have a type of enclosure or that the specific data were not obtainable. Grey cells in support type and orientation indicate there were no supports of that category (rigid/ flexible/ other) in the specific enclosure. Full explanation is given in the Methods.

Fig 2 shows the five Enclosure Complexity Indices we created to translate the concepts of support complexity, diversity and temporal change from natural to captive habitats to provide an overall complexity profile of each enclosure type for each zoo. The highest scores were for the frequency of changes over time; one modified all three enclosure types at least once per year; one modified two at least once per year, three changed some components in one enclosure at least once per year and two only made changes where furniture broke. For the ratio of flexible supports to rigid; one zoo scored complex (flexible supports > rigid supports) or moderate (flexible and rigid supports equally available) for two enclosure types; three scored at least moderate for one enclosure and three scored simple (rigid support > flexible supports) for all enclosures type available. For density; two zoos had high (complex) density and two scored moderate density for the indoor enclosure, one scored moderate for off-show and one scored moderate for outdoor. The rectangles in Fig 2 show that the zoos were mostly providing at least moderate diversity of rigid and flexible support types and orientations, but fewer items that could be used for weight bearing such as barrels and tyres. Overall, the ECI’s found meaningful variation between the zoos in habitat complexity, but measure of density needs refinement since it need to show how density varies within as well as between spaces. Moreover, our density scores also did not account for the fact that some supports are long and some are short. Further consideration of optimal support density in the different enclosure types may need to include several measures from each enclosure, or perhaps a measure of how much of the enclosure volume allows sustained off-ground locomotion.

In a basic resource-based welfare assessment, the presence of arboreal climbing frames in the outdoor and indoor enclosure would be taken to indicate that the zoos were providing adequately for the arboreal needs of the chimpanzees. However, the Enclosure Complexity graphic translates multiple strands of information on enclosure furniture to create a visual overview of complexity relative to wild habitats and indicates that there is substantial capacity to improve habitat complexity, even in small spaces, by increasing the number of flexible supports, varying support types and orientations and changing the enclosure furniture on a regular basis. This aspect is particularly important since zoos need to offset reductions in available space during the winter to allow natural relationships and behavioural profiles. This process adds considerable detail to an assessment of the extent to which zoos are ensuring that chimpanzees can show natural movements and experience sufficient biomechanical variation to maintain normal musculoskeletal function and development.

Another key aspect of habitat complexity is nest building. Wild chimpanzees are known to construct sophisticated and elaborate nests for sleeping during the day and night in trees [83]. As wild chimpanzees spend half of their time in nests, this should be viewed as an essential component of their environmental requirements in captivity [29]. Nest building itself is a complex behaviour, requiring a sequential combination of bending and breaking branches into a secure, safe, comfortable platform [84,85]. Captive chimpanzees, like their wild counterparts, also seem motivated to build nests [86,87], but early rearing conditions, including whether infants are maternally -reared, influences nest building skills [88]. Captive chimpanzees spend a similar amount of time building nests as wild chimpanzees (1–5 minutes [89,90]). They use various materials [91,92] and, when provided with nesting structures, substrates and varied materials, express species-typical behaviours such as manipulation and exploration (e.g., [86,87]). Captive chimpanzees most frequently construct nests on elevated platforms, tunnels and nesting baskets, reflecting the arboreal pattern typical of their wild counterparts [90,93].

In this study all zoos provided nesting materials during both the day and at night. Two delivered more than four different nesting materials, including two that provided up to six different materials (see S2 Results). Five zoos provided between 1–3 materials. The materials included hessian sacks, sheets, old clothes, duvets, woodwool, plant material, paper, straw, haylage, hay and browse. However, of these, only browse might realistically create the opportunities needed for natural, complex bending and weaving behaviours, when the nest base allows attachment points to secure branches so the chimpanzees can bend and weave against a firm base, mimicking the green stick fractures used by wild apes [85]. When these are provided studies have shown that captive chimpanzees will adopt more natural nesting behaviours [25].

Shelter is the final component of habitat complexity. There are ample sources of shelter in most natural habitats, which also provide visual and physical barriers between individuals. In captivity shelters not only provide opportunities to retreat from weather, they also allow the apes to be out of sight of visitors and each other. This is a major component of their ability to make meaningful choices and when absent can be a source of significant distress (e.g., [94]). In this study two zoos provided outdoor shelter under which all chimpanzees could take cover at the same time, the remaining 7 provided multiple caves, climbing frames and vegetation under which a smaller number of individuals could shelter at any one time. In general indoor spaces were accessible as well.

### Feeding and foraging

In the natural habitat chimpanzee food sources are unevenly distributed, both physically and temporally. Wild chimpanzees must find ripe fruits within a territory of at least 20 km^2^ in which fruiting trees are widely scattered, their production is highly seasonal, and visibility is often limited to about 30 meters. They also use complex foraging techniques as many of their food items require manual processing or extraction with a tool (e.g., [95]). The physical and cognitive challenge of finding and accessing food are therefore substantial. Food availability can be extremely variable between different habitats and this likely explains the variation in the time wild chimpanzees spend feeding and foraging; typically, between 20% and 45% of their waking time [96–99].

Two important considerations for captive chimpanzees are whether food is provided at the same times each day and whether each individual’s intake is controlled, since managing access to food is a common practice to tackle the risk of increased obesity and related diseases (e.g., [100]). Studies on these topics are limited. Bloomsmith and Lambeth’s [101] study indicated that an unpredictable schedule for main feeds reduced inactivity in laboratory-housed chimpanzees compared to a predictable schedule, where the chimpanzees seemed to be waiting for their food to arrive. The effect, however, was small as food-based enrichment was still scheduled and monkey biscuits were always available. Howell & Fritz [102] reported higher pre-feeding levels of agonism in some captive chimpanzees experiencing predictable, compared to unpredictable, feeding schedules. Studies across a range of species have shown that unpredictable schedules for positive events like feeding are associated with signs of better welfare, such as lower abnormal behaviours and aggression, although signal reliability is thought to be important [103,104]. In addition, restricted feeding has been shown to correlate positively with abnormal behaviours (e.g., review by Hill [105]). Further research is needed to establish whether the potential negative effects of predictable and controlled feeding schedules outweigh the positive benefits of enhancing the health of captive chimpanzees. However, it is noteworthy that no studies to date have indicated that a predictable feeding schedule is preferable to promote welfare. Moreover, the welfare benefits of both techniques are compromised by the fact that the chimpanzees have no control or agency over the acquisition of food or feeding times and durations.

A related issue is that wild chimpanzees travel a minimum of 2–4 km daily and can spend half of their day feeding [96–99]. While zoos should not necessarily aim to match frequency of behaviours to the wild, providing variable methods of food delivery can increase feeding and foraging times, which reduces inactivity and boredom and contributes to controlling excessive caloric intake [29]. Searching for food items that are widely dispersed (i.e., scattered or patchy distributed) tends to occupy longer periods of time than consuming items fed in a single location [106], but searching itself in captivity is often not a mentally challenging activity. In contrast, feeding chimpanzees off the ground, particularly high up in the enclosure, mimics foraging in the trees and encourages exercise and natural locomotor behaviours [25]. Varying foods temporally can mimic natural seasonal variation [93]. Moreover, increasing the time chimpanzees spend engaged in complex foraging techniques, such as tool use, provides cognitive challenge and environmental enrichment [102,107,108]. While there is little current evidence regarding the optimum number of daily meals chimpanzees should be fed for good welfare, encouraging activity by providing a highly complex environment with self-feeding devices and hiding food off the ground should greatly boost their health and mental state through greater control and choice over when/where to feed.

In this study access to food was dependent on enclosure access, enrichment provision, and feeding times. Three zoos had a written Feeding and Nutrition Plan that accounted (to some extent) for each individuals’ age and nutritional requirements and included regular monitoring of intake (Table 3). Two had no formal plan and did not account for the nutritional demands of the different ages and individuals, although one did monitor intake. The numbers of feeds per day varied widely (from 3–10). All zoos described that they employed feeding strategies that were sensitive to dominance hierarchies, such as scattering food to give all individuals the opportunity to find it, targeted feeding, patchy distribution across the enclosure and arboreal feeding. Only one zoo varied the times of feeding. Table 3 shows that all zoos used scatter feeding and food-related enrichment devices on a daily basis. The number of different feeding techniques used on a daily basis ranged from 3–8. Interestingly, zoos without a Feeding and Nutrition plan utilised more feeding techniques than those with a formal plan; the three zoos with a Feeding and Nutrition Plan provided a mean of four (SD: 2.00) techniques per day and had three (SD: 2.65) that were never provided, compared to those with no plan who provided five (SD: 1.63) techniques daily and had three (SD: 0.82) that were never provided. This highlights the core concept that formal plans are only as good as the implementation. Zoos that lack sufficient resources to execute formal plans may deliver lower welfare than those with more resources but no formal plan; which again highlights the need for resource-based welfare assessment tools to be detailed and integrated.

**Table 3:** Feeding planning, management and provision by the zoos.

Zoo	1	2	3	4	5	6	8
**Feeding and Nutrition Plan that:**	N	Y	Y	N	Y	N	N
accounts for age	N	S	Y	S	Y	S	N
accounts for individual nutritional requirements	N	S	Y	S	Y	S	N
includes monitoring	N	S	Y	S	Y	S	S
**Times fed per day**	3	4	6-8	10	4	6	3
**Fed at same time per day**	Y	Y	N	Y	Y	Y	Y
**Distribution of preferred foods accounts for dominance**	Y	Y	Y	Y	Y	Y	Y
**Distribution of balanced diet accounts for dominance**	Y	Y	Y	Y	Y	Y	Y
**Types of food provision**^**a**^:							
Scatter feed	daily	daily	daily	daily	daily	daily	daily
Patchy distribution	daily	>1 week	daily	daily	never	daily	never
Hand fed	daily	daily	daily	>1 week	daily	>1 week	>1 month
Controlled feeding	never	never	< 1 month	never	never	never	never
Self feeder	never	never	never	never	never	never	never
Arboreal feeding	daily	daily	< 1 month	daily	never	daily	daily
Hidden arboreally	daily	daily	< 1 month	never	never	daily	>1 week
Hidden in vegetation	daily	daily	daily	daily	>1 week	never	daily
Hidden in substrates	daily	daily	>1 month	daily	never	daily	never
Enrichment devices	daily	daily	daily	daily	daily	daily	daily

Y: Yes; N: No; S: Somewhat.

^a^The question regarding types of food provision allowed 5 responses, so the colours indicate very frequent (pale blue), frequent (pale green) moderately frequent (mid blue), rare (dark green), and never (dark blue). Parameters are not colour coded if the evidence for welfare is not yet established. Scatter feed is coloured grey because it has aspects of low and high welfare: low because it is not a cognitively or physically challenging feeding technique, but high because it can be used to account for dominance and the need for a balanced diet.

### Choice and control

Positive animal welfare is effectively characterized by four features: positive emotions; positive affective engagement; quality of life; and happiness [109]. However, assessing mental experience and welfare measures are notoriously difficult because they are felt by the animal and are thus subjective. Littlewood and colleagues [10] recently argued that the way to resolve this issue is animal agency, defined as the capacity of animals to engage in voluntary, self-generated, and goal-directed behaviour that they are motivated to perform [110]. This approach is implicit in the 5 Domains Model of animal welfare [9], but its importance was made explicit in the renaming of Domain 4 in 2020 to ‘behavioural interactions’, which clarified that this domain represents the animal’s ability to exercise agency in their interactions with the environment, other animals, and humans [111].

Choice and control are interrelated aspects of exercising agency [10]. Zoo chimpanzees have control when they can consistently and predictably make choices and obtain the outcomes they are motivated to achieve (e.g., [112,113]); which can be in interactions with their environment, other animals, and humans. Inaction is as essential as action; a chimpanzee choosing not to interact (e.g., the option to not take part in positive reinforcement training) exerts control over its actions and therefore exercises agency [112,114]. Since chimpanzees are highly intelligent and have very different individual personalities, it is difficult to predict the behaviours that individuals may want to make choices about. A wide variety of opportunities to make decisions therefore needs to be available. Although, the basis should be in the animal’s natural behavioural ecology, zoo settings can offer opportunities for decision making that are not available in natural habitats, such as using technologies [9,10].

**Fig 3:**
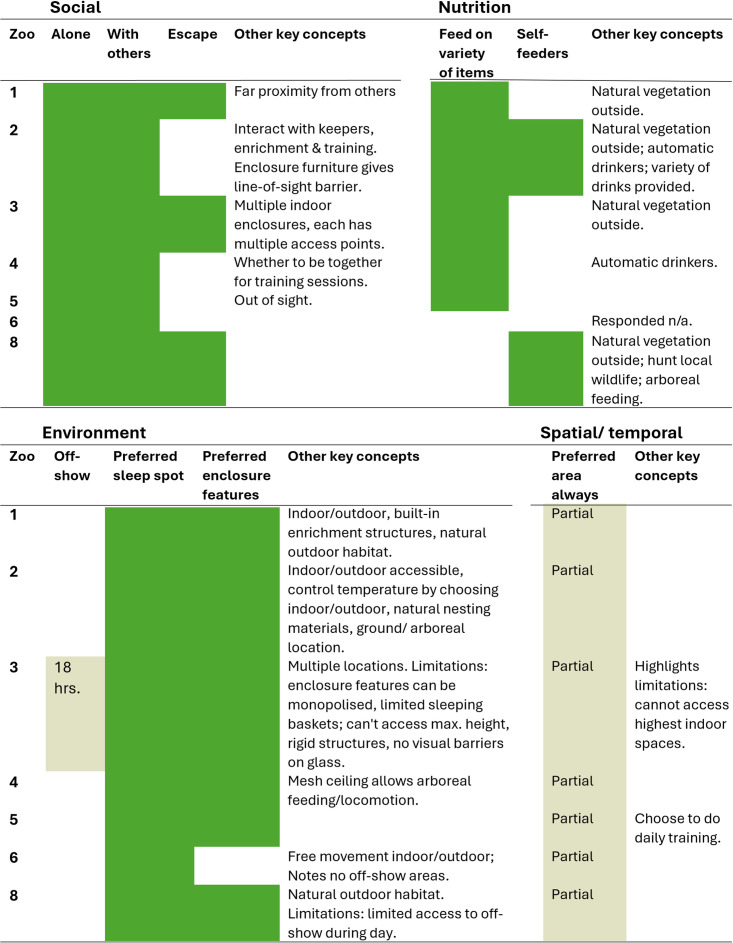
Aspects of the chimpanzees’ lives where they can express choice and control. Aspects in the separate columns were provided as examples in the survey and green shading indicates zoos that highlighted these as present for their chimpanzees. Aspects in the final column were free text comments provided by respondents. Green shading is used (rather than the blue in other tables) because it reflects what the zoos report is present rather than an indication of welfare quality.

In this study zoos were asked to describe the ways in which the chimpanzees could exert control over social, environmental, nutritional and spatial/temporal aspects of their lives; some examples were given in the question for each category. Fig 3 shows where zoos directly reflected those that were provided as examples and where addition keywords/concepts were provided independently. Although zoos were not specifically asked to identify limitations in giving the chimpanzees control over their lives, some added these in their responses. For social aspects, all considered that their chimpanzees could choose to be alone or with conspecifics and five specified that chimpanzees could separate from other chimpanzees if needed. Additional opportunities included choices about individual or social training, objects for use in male display and being out of sight. For nutrition five considered that could feed on a variety of food items but only two referred to self-feeders. Additional opportunities predominantly related to feeding on natural resources outside and one cited hunting local wildlife. For environmental aspects, all felt their chimpanzees could choose where to sleep at night and most noted they could interact with preferred enclosure features. Most additional opportunities related to choosing to be indoor or outdoor, several highlighted lack of opportunity to be off-show as a limitation. Finally for spatial and temporal, no zoo felt that their chimpanzees could always access all preferred areas. Most specified the limitations on access for different spaces (similar to the results presented in Table 2).

### Review of the effectiveness of the AWARE Tool

An underpinning concept has been that the tool should be based on the extent to which resources and husbandry facilitate the expression of natural behavioural ecology. There are several potential methodological and theoretical problems with a focus on emulating natural behaviours in zoo settings (see [115); the two primary ones are that a difference between behaviour in captivity and in the wild does not necessarily imply that animals in captivity have poor welfare and that animals in the wild may also have poor welfare under some circumstances and thus cannot be used as a benchmark for good welfare [116]. Nevertheless, if zoos are to fulfil their educational and conservation roles, they must hold animals whose behaviour represents their wild counterparts to the best extent possible. Moreover, musculo-skeletal and mental health are clearly linked to the expression of natural behaviour [16,25,116]. Here, we have focussed on ensuring that enclosure furniture and husbandry allows, but does not limit the animals to, the expression of natural behaviours. We argue that the presence of this option is critical to achieve positive welfare, even though actual behavioural expression may not emulate natural behaviours as it will be influenced by individual preference, group cultures, coping mechanisms, adaptation to captivity and the fact that chimpanzees are generalists and extremely flexible in their behaviour [117]. This approach is consistent with the dynamic animal welfare concept (DAWCon, [11]) which focuses on the dynamics of an animal’s environment over time, and the animal’s behaviour in response to changing environmental conditions. It argues that animals should have good welfare when they have the mental and physical ability and opportunity to react to the net effect of positive and adverse stimuli, events and conditions over time [11]. Nevertheless, a weakness in the model is that just because an animal can cope or adapt to a negative environment doesn’t mean its welfare is good. There is a risk of confusing resilience with wellbeing since captive animals can show behavioral and physiological signs of adjusting to chronic stress or poor environments (e.g., small cages, lack of enrichment), but this doesn’t mean they are thriving. Adaptation does not equal happiness or a high quality of life. While DAWCon highlights that behavioral flexibility is important, positive welfare must go beyond coping and include avoiding chronic stress (even if the animal seems to cope), environments that promote thriving (not just surviving), opportunities for choice, and positive emotional experiences.

The results show the AWARE Tool proved effective in revealing nuanced differences in how captive environments support or constrain chimpanzee wellbeing that would not have been detected through conventional resource-based approaches alone. By integrating detailed information on group composition, space use, habitat complexity, feeding practices, and opportunities for choice and control, the tool enabled a holistic evaluation of how social, physical, and management factors interact to shape individual welfare outcomes. Its capacity to link input-based indicators (e.g., enclosure dimensions, group size) with outcome-based indicators (e.g., opportunities for social choice, expression of natural behaviour) demonstrated that apparent adequacy in one domain can be undermined by deficiencies in another. Moreover, the tool’s combination of quantitative data with qualitative insights on behavioural opportunities allowed comparisons between zoos while accounting for contextual variability. This can highlight, for example, where welfare potential can be enhanced in smaller spaces through greater structural complexity and variability, or compromised in larger spaces when access and agency are limited. Overall, the tool provided a sensitive and integrative framework for identifying both strengths and welfare risks within captive management systems, demonstrating its practical value for evidence-based welfare improvement and adaptive enclosure design.

In terms of completion, zoos found the self-assessment stage to be easy to complete. Although completion of the second stage was supported in person by one of us (JN), it would be possible in the final iteration of the tool to provide online guidance to ensure the questions are completed accurately. Analysing the survey responses identified questions that worked well and those that didn’t for chimpanzees. The combination of juveniles and adolescents into a single ‘immature’ category made it difficult to draw any nuanced conclusions on age-related welfare for younger individuals, so in future versions these age groups should be separated. Some questions on attitudes, such as ‘Do you perceive these different practises to be routine husbandry or enrichment’ (S1 Survey, Q5.5.8), proved less informative than we predicted, and were omitted from the analysis. Some were found to require more information; such as the question on barriers used in the enclosures (S1 Survey, Q2.11) where it would have been useful to have obtained a measure of the amount of wall and ceiling space covered by each material (particularly the amount of mesh covering to reveal the extent of the chimpanzees’ ability to utilise their 3D space). Some questions had phraseology that seemed to be interpreted slightly differently by different zoos; such as the confusion in responses to the question regarding how often zoos assess welfare (S1 Survey, Q5.1). Similarly, the survey asked if zoos had an Enrichment Plan, which they appear to have interpreted as a written plan since most said they did not but their narrative responses indicated they had routine enrichment procedures. We tackled whether the chimpanzees had opportunities to make social choices or be alone in several ways, asking: if all apes have the opportunity to be alone or in far proximity (>5m) (S1 Survey, Q4.1); the maximum number of individuals that are housed in each room regularly (S1 Survey Q2.4); and whether all apes can access all enclosures spaces at all times (S1 Survey, Q2.1). The latter proved to be the most informative (and is the one we selected to present) as it employed a multi-enclosure approach and the usage component of the question related to routine husbandry and zoo policies on weather rather than relying on keeper observations. Clearly there is a risk of error in questions that rely on keeper observations as the amount of time they have to observe their chimpanzees differs between zoos and responses will be anecdotal rather than scientifically guided. Nevertheless, qualitative questions can be phrased in a way that provides useful baseline guidance on, for example, the minimum extent to which individuals can exploit their environment, which can highlight areas that need further analysis by the organisation. For example, the question on whether all age classes could utilise all available heights (S1 Survey Q2.5.1 ) indicated important constraints on height use by elderly individuals. In refining the survey, we also need to further innovate and expand ways to quantify the apes’ ability to have agency over their lives.

### Validation

**Fig 4:**
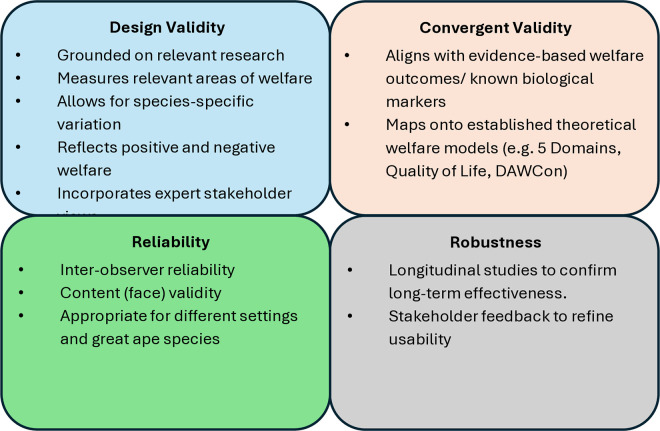
The validation framework for welfare assessment tools.

Validating a holistic welfare assessment tool for zoo-housed great apes is complicated by their social, behavioural and ecological complexity; the need to be non-invasive, and the fact that it is neither informative nor feasible to compare each individual measure against one or more benchmark welfare indicators, where such benchmarks even exist. Validation must therefore occur via the collection of multiple lines of evidence that examine the tools scientific and convergent validity, reliability and robustness. We propose that the framework in Fig 4 (building on [115] and [118]) achieves this. The first component (design validity) is critical to ensure a welfare assessment tool is scientifically valid through grounding the selection and nature of measures on the behavioural ecology, psychology and welfare of all relevant species in wild and captive settings; complementing this data with unpublished expert knowledge from practitioners and other stakeholders, and ensuring the tool can reflect both positive and negative welfare states. The second component (convergent validity) focuses on ensuring that the selected measures map onto the existing evidence base for each species, such as key welfare models and positive welfare outcomes. A welfare assessment tool needs to be reliable (component 3) in the sense that it can be implemented consistently in different settings, by different users and for different species. Ensuring that the individual questions really ask what they are meant to be asking (face validity) is also a key part of this component. Finally, the welfare assessment tool needs to be robust (component 4) such that it reveals variation in welfare over time or due to changes in resources and husbandry and is considered to be valuable by end users and other stakeholders.

For the AWARE Tool we have completed Components 1 and 2 for all great apes: selection of the measures and interpretation of the results were both based on the research literature on great ape behavioural ecology and welfare, including underdeveloped areas such as agency and competence. We also incorporated the wide range of expertise of colleagues on the Great Ape Welfare group, including academics, zoo practitioners and representatives from DEFRA and the RSPCA. The AWARE Tool maps onto the 5 Domains Model of animal welfare: resource-based measures are well aligned with Domains 1–3 of the model due to their input focus. In the renaming of Domain 4 to behavioural interactions [111], three subcategories were included to encourage users to consider opportunities for animals to exercise agency during interactions with: (A) the environment; (B) other animals; and (C) humans. To achieve this, we employed an augmented approach by combining a wide range of resource measures with usage information and narrative responses and the integrated analysis of resource and usage responses to create nuanced interpretations of the capacity for behavioural expression (such as the Enclosure Complexity Indices). We have confirmed reliability for chimpanzees. Papers for gorillas and orangutans are in progress, which will lead to final refinements to the survey to ensure face validity and its application to all species in all settings. The final components of validation will be to repeat the survey in several years to establish its long-term effectiveness and to consult with end users to ensure the final version of the AWARE tool meets stakeholder needs. Nevertheless, the results suggest that our approach enhances resource-based welfare assessments to provide a nuanced and meaningful understanding of great ape welfare, whilst allowing data collection within a short space of time.

### Welfare recommendations and progress

Several recommendations for the care of chimpanzee groups are clearly indicated by this survey, remembering that the AWARE Tool sought to enhance welfare provision beyond levels required by inspection and accreditation standards. Firstly, group composition has a major impact on welfare, particularly since youngsters increase the welfare of the whole group and the selection of social partners is a key area where they can exercise agency. The unbalanced age structure for chimpanzees in UK zoos is beginning to resolve since there were 14 births from February 2020 – December 2024 compared to just 3 in the 5 years preceding the survey (although it is possible that not all the recent births survived [119]). Enclosure complexity is generally under-developed, and zoos can easily increase the chimpanzees’ physical and mental challenges, natural feeding, social choice, options for separation/avoidance, and musculo-skeletal health, even in small spaces, following the concepts underpinning the Enclosure Complexity Indices. For new enclosures the inclusion of mesh as a barrier is also recommended as it optimises the chimpanzees’ ability to utilise all of their 3D space, makes it easy to change locomotor structures over time, and allows escape pathways for subordinate animals.

We also show that space is valuable in itself, and zoos should aim to expand enclosures both horizontally and vertically whenever possible, as increased activity and space usage likely increase disproportionately with added space. Increasing enclosure complexity, creating multiple defined areas and adding environmental enrichment to give the chimpanzees a reason to exploit underused areas can also substantially improve welfare, if well planned and matched to the needs of each individual.

We encourage the use of written plans for feeding, welfare, healthcare and environmental enrichment, that are targeted to address specific issues relevant to each group so that zoos can ensure a strategic and consistent approach that can ensure changes of staff do not impact on the continuity of care. However, these plans also need to be monitored and realistic, so they are responsive to changes in the individual’s welfare state and achievable within the zoo’s daily practises. This is an area that zoos have been progressing since the survey took place, led by zoo associations’ increasing requirements for such things to be in place. Understanding of areas in which zoo housed chimpanzees can make choices and have control over their lives is underdeveloped. BIAZA and the Great Ape Welfare group (GAWg) should lead in developing discussions to support zoos to better understand how they can maximise the chimpanzees’ agency over their lives.

Finally, there are crucial gaps in knowledge in key aspects of chimpanzee behavioural ecology and captive welfare. There is considerable capacity for a stronger collaboration between science-led projects and the zoo community to improve the health and welfare of zoo-housed chimpanzees. Again, BIAZA and GAWg should play a central role in closing this gap.

The zoo sector continually develops its animal care and a number of changes in their approach to animal welfare have occurred since the survey that are relevant to highlight here. The World Association of Zoos and Aquariums introduced animal welfare goals for all of its members to implement. These include compliance with an animal welfare evaluation process implemented by a relevant national or regional zoo association. This evaluation process must be based on the five domains model of animal welfare and include both input and output, positive and negative indicators of welfare. BIAZA, as the national association for where this survey was conducted, subsequently updated their welfare policy, requiring all members to conduct formal animal welfare audits (BIAZA 2023), and introduced a formal membership accreditation programme. These steps will undoubtedly have positive impacts on the welfare of chimpanzees cared for by zoos.

The zoos involved in this study have also made major advancements in chimpanzee welfare since the study: at least two are in the design phase for new enclosures. Others now provide 24/7 access to all indoor, outdoor and off-show spaces simultaneously, or have built additional spaces. They have increased habitat complexity and the number/diversity of locomotor supports that can be changed, and made changes to indoor viewing arrangements so chimpanzees can be out of view of visitors – high up or behind visual barriers/vegetation. Several have instigated welfare assessments/audits for chimpanzees and/or all species in their care, and other have increased welfare monitoring. They also report expanding daily enrichment provision related to the five enrichment categories; the diversity of nest building materials, reviewing/expanding nutrition plans and expanding feeding diversity, especially related to arboreal feeding. Several have expanded training plans and increased the extent of behaviours included in positive reinforcement training. Many reported developing targeted welfare support for geriatric individuals, including operant training for monitoring age-related conditions and bespoke welfare/dietary assessments. Moving forward, the Great Ape Welfare Group (GAWg) will lead on reporting the results of the study back to the community and providing targeted training and guidance to help all chimpanzee-holding zoos apply these results to continue to work towards their own aspirational levels of welfare.

## Conclusion

This study aimed to provide a holistic and informative assessment of the variation in welfare provision of chimpanzees at different zoos by: collecting a holistic and integrated suite of resource and husbandry data that optimised the information gathered about potential behavioural expression; embedding the results in the research literature on wild and captive chimpanzee behavioural ecology and welfare to provide a nuanced and meaningful understanding of how resources and husbandry can translate into behavioural expression. We included components that exceed the level of welfare required by the zoo inspection and accreditation processes and Best Practise guidelines, so that each zoo can use the tool to achieve the levels of welfare they aspire to. Our approach acknowledges the complexities of assessing welfare in captive great apes, balancing theoretical considerations with practical applications that allow for dynamic interpretation across different settings and species.

The results show that incorporating dynamic measures (such as minimum accessible space under different management or weather conditions, the diversity and flexibility of enclosure supports, and opportunities for chimpanzees to make meaningful choices) into resource-based assessments provides a more accurate reflection of the lived experience of individuals than those that rely on resource based measures (e.g., sufficient group size or enclosure area). The sensitivity of the AWARE tool in capturing these multidimensional interactions therefore represents a critical advance toward identifying both the potential and limitations of captive environments in supporting optimal chimpanzee wellbeing.

Validation of the AWARE Tool has been undertaken through a multi-component framework addressing design, convergent validity, reliability, and robustness. Results for chimpanzees indicate that the tool successfully integrates resource usage with behavioural ecology principles, providing a meaningful and scientifically grounded welfare assessment. Further research and refinements for gorillas and orangutans are underway, ensuring the tool’s applicability across all great ape species and settings. Long-term validation efforts, including repeat surveys and stakeholder consultations, will further enhance its reliability and practical utility. Ultimately we aim for the AWARE Tool to be an automated tool that can analyse and report on each zoo’s uploaded results, linking outcomes with recommendations to improvements.

Key welfare recommendations emerging from this study emphasize the importance of group composition, enclosure complexity, and opportunities for agency. Addressing these areas, alongside structured welfare planning and evidence-based management strategies, will enhance welfare outcomes across zoo-housed great apes. Encouragingly, since the survey’s completion, substantial advancements have been observed in zoo practices, reflecting an ongoing commitment to improving great ape welfare.

The role of BIAZA and GAWg will be pivotal in supporting zoos to implement these findings effectively. Future collaborations between scientific research and zoo management will further bridge gaps in understanding and enhance welfare standards. The AWARE Tool provides a valuable tool for assessing and improving great ape welfare, contributing to the ongoing evolution of best practices in the zoo sector.

## Supporting information

S1 Survey Survey. (DOCX)

S2 File Additional Results and references. (DOCX)
