# Supplementary material for: Developing and validating a holistic welfare assessment tool for zoo-housed great apes: Integrating resource-based measures with behavioural ecology insights
Source: PLoS One. 2026 Jan 30;21(1):e0340094. doi: 10.1371/journal.pone.0340094 (PMC12858012; doi:10.1371/journal.pone.0340094)
Supplement: S1 Survey — (DOCX) [file pone.0340094.s001.docx]

Supplementary survey 1

Facilities & Management Survey

- Part I –

**This is survey part I, which contains blue-highlighted questions that can be answered by the zoo in advance (but I am also happy to help with these during the visit).**

**Please complete one survey document per species.**

**Please note, if you hold multiple groups of the same species in a single facility but with no shared space this will be considered multiple facilities and you should complete a different questionnaire for each facility.**

**If you hold all groups of the same species that rotate through the same enclosure spaces this would be treated as one facility, so one questionnaire would be answered but please code the groups G1, G2, G3 etc.**

**Interpretation of terms used**

The following terms as defined here are used in this zoo facility and management survey.

- **Indoor enclosure:**  Publicly-viewable inside area of an exhibit.
- **Outdoor enclosure:** publicly-viewable outside area of an exhibit.
- **Off-show areas:** Unless specified this term refers to the primary off-show areas that apes regularly use, rather than secondary off-show areas which are rooms used for specific purposes (such as quarantine, introductions, hand-rearing, medical treatment, specialized research observation, etc.).
- **Enclosure barrier:** The physical barrier that contains the animals in the enclosure.
- **Visual barrier:** Visual barrier that enables animals to hide from public view and/or be out of sight of conspecifics, but may be made from flexible material.
- **Three-dimensional off-ground locomotor structures:** Vertical, horizontal and angled pathways with cross-connections between them at variable height levels.
- **Positive reinforcement:** The introduction of a desirable or pleasant stimulus (i.e., a reward) after a behaviour. The desirable stimulus reinforces the behaviour, with the intention of making it more likely that the behaviour will occur again.

**Individual details**

|  | **House name** | **Age** | **Sex** | **Group number** (G1, G2 etc.) |
| --- | --- | --- | --- | --- |
| **1** |  |  |  |  |
| **2** |  |  |  |  |
| **3** |  |  |  |  |
| **4** |  |  |  |  |
| **5** |  |  |  |  |
| **6** |  |  |  |  |
| **7** |  |  |  |  |
| **8** |  |  |  |  |
| **9** |  |  |  |  |
| **10** |  |  |  |  |
| **11** |  |  |  |  |
| **12** |  |  |  |  |
| **13** |  |  |  |  |
| **14** |  |  |  |  |
| **15** |  |  |  |  |
| **16** |  |  |  |  |
| **17** |  |  |  |  |
| **18** |  |  |  |  |
| **19** |  |  |  |  |
| **20** |  |  |  |  |
| **21** |  |  |  |  |
| **22** |  |  |  |  |
| **23** |  |  |  |  |
| **24** |  |  |  |  |
| **25** |  |  |  |  |

| **Institution** |  |
| --- | --- |
| **Support**  **Name**  **Role** |  |
| **Surveyor(s)**  **Name 1**  **Name 2** |  |
| **Date/ Time** |  |

1. **Species**

| **Species** |  |
| --- | --- |

- 1. How many groups of this species do you manage?

|  |
| --- |

- 1. Do these groups “share” (rotate through) enclosure spaces? Please describe:

|  |
| --- |

1. **Enclosure**

**Note: Primary off-show areas are areas off-show to the public that apes access regularly (e.g., sleeping dens). Secondary off-show areas are areas off-show to the public that are only used in exceptional circumstances (e.g., for introductions).**

- 1. Can all your apes access all indoor, outdoor and primary off-show areas at all times during the day and night (except to allow short periods of routine husbandry)? Please include details if there are periods when they don’t have access, which may include, for example seasonal variation, new births, elderly or ill individuals, etc.

| **Daytime** | **Yes** | **No** | **Somewhat** | **Notes** (If no, somewhat, please give details on group number and reason) |
| --- | --- | --- | --- | --- |
| Indoor |  |  |  |  |
| Outdoor |  |  |  |  |
| Primary off-show |  |  |  |  |
| **Overnight** | **Yes** | **No** | **Somewhat** | **Notes** (If no, somewhat, please give details on group number and reason) |
| Indoor |  |  |  |  |
| Outdoor |  |  |  |  |
| Primary off-show |  |  |  |  |

- 1. Does the facility have secondary off-show areas (see definition section above)? If yes, please explain below what they are used for and indicate how frequently they are used.

|  |
| --- |

- 1. Which structures in your enclosure can be changed (e.g., location, orientation)? Please list:

| **Indoor** |
| --- |
| **Outdoor** |
| **Primary off-show** |

1. **Environmental Parameters**
   1. Is there artificial UV provision?

| **Yes** |  |
| --- | --- |
| **No** |  |

- 1. If yes, how often are readings taken and what are they?

|  | **Daily** | **Weekly** | **Monthly** | **Yearly** |
| --- | --- | --- | --- | --- |
| **Frequency** |  |  |  |  |
| **Latest measurement of UV light intensity** |  | | | |

- 1. Do indoor areas have UV permeable ceilings/windows? If yes, give the area of the UV permeable ceilings/windows.

|  |
| --- |

- 1. What is the general temperature that your indoor enclosure and primary-off show areas measure?

| **Indoor enclosure** (all rooms combined) |  |
| --- | --- |
| **Primary off-show area** (all rooms combined) |  |

- 1. Do you use mechanisms to reduce the apes’ exposure to undesirable noise (e.g., visitors, machinery, heaters/fans, or other animals)? If yes, what do you use successfully? Please explain below.

|  | **Noise source** | **Mitigation** |
| --- | --- | --- |
| **Indoor** (all rooms combined) |  |  |
| **Outdoor** (all rooms combined) |  |  |
| **Primary off-show** (all rooms combined) |  |  |

**3.6** What mechanisms do you use successfully to reduce the visual impact of visitors? Please list below:

| **Indoor** |  |
| --- | --- |
| **Outdoor** |  |

**3.7** How frequently do you make any changes to the enclosure? Are changes *ad hoc* or guided by a Management Plan?

| **Enclosure** | **Rarely/ only when features break** | **Occasionally (> once per year)** | **Often (> 4 times per year)** | **Notes** (including how much of enclosure is modified each time)**:** |
| --- | --- | --- | --- | --- |
| **Indoor** (all rooms combined) |  |  |  |  |
| **Outdoor** (all rooms combined) |  |  |  |  |
| **Primary-off show** (all rooms combined) |  |  |  |  |

1. **Social grouping**
   1. Do all your individuals have a daily and nightly opportunity to spend time physically alone or in far proximity (> 5m horizontally and/or vertically) to others?

| **Daytime** | **Yes** | **No** | **Notes** (If no, please explain) |
| --- | --- | --- | --- |
| Indoor |  |  |  |
| Outdoor |  |  |  |
| Primary off-show |  |  |  |
| **Overnight** | **Yes** | **No** | **Notes** (If no, please explain) |
| Indoor |  |  |  |
| Outdoor |  |  |  |
| Primary off-show |  |  |  |

**4.2** Do you ever separate any individuals from their group for short periods of husbandry? If so, please provide details below (e.g., G1: reason, G2: reason, etc.).

|  |
| --- |

**4.3** During routine separation can separated individuals remain in contact with conspecifics?

|  | **Yes** | **No** | **Notes** (if no, please explain why) |
| --- | --- | --- | --- |
| **Physical** |  |  |  |
| **Visual** |  |  |  |

**4.4** Does your facility have any of the following features to aid in introductions? Tick all the relevant boxes.

| **Facility features** | |
| --- | --- |
| **Howdy cages** |  |
| **Introduction cages** |  |
| **Hydraulic or ratchet slides** |  |
| **Crush cages** |  |
| **Races** |  |
| **Bare cages** |  |

1. **Behavioural Management**
   1. Do you undertake welfare assessment of this species? If so, how often? What time of the day? How long for per animal? Is it guided by an assessment tool? Are independent observers involved?

|  | **Notes** (specify here): | | |
| --- | --- | --- | --- |
| **How often?** |  | | |
| **What time of the day?** |  | | |
| **How long for per animal?** |  | | |
|  | **Yes** | **No** | **Notes** (If no, give reason why): |
| **Is it guided by an assessment tool?** |  |  |  |
| **Are independent observers involved?** |  |  |  |

- 1. Do you have a written Welfare Plan that guides the assessment of positive and negative welfare states for behavioural, physical, physiological and psychological measures, and mitigation plans for any welfare issues? Tick only those for which the answer is yes.

|  | **Behavioural** | **Physical** | **Physiological** | **Psychological** |
| --- | --- | --- | --- | --- |
| **Positive welfare** |  |  |  |  |
| **Negative welfare** |  |  |  |  |
| **Mitigation plans** |  |  |  |  |

- - 1. If yes, please complete the table below and list the positive and negative indicators that your welfare plan includes for behaviour, physical, physiological and psychological measures. Alternatively, please can we have a copy of your welfare plan?

|  | **Behaviour** | **Physical** | **Physiological** | **Psychological** |
| --- | --- | --- | --- | --- |
| **Positive welfare indicators** |  |  |  |  |
| **Negative welfare indicators** |  |  |  |  |

**5.3** Who is responsible for/involved with planning, implementing and monitoring welfare measures for the species?

|  | **Research team** | **Welfare Officer** | **Keeper** | **Senior Management** | **Team leader** | **Curator** |
| --- | --- | --- | --- | --- | --- | --- |
| **Planning welfare** |  |  |  |  |  |  |
| **Implementing welfare** |  |  |  |  |  |  |
| **Monitoring welfare** |  |  |  |  |  |  |

- 1. Do you have an acquisition, transfer, & transition policy that reflects commitment to prioritizing the welfare of individual animals during their lifetimes (e.g., prohibits transfer to situations that would compromise welfare)?

| **Yes** | **Somewhat** | **No** | **Notes** (If no, somewhat, please explain) |
| --- | --- | --- | --- |
|  |  |  |  |

**5.5** Do you have an Enrichment Plan? Does it address defined behavioural goals and include evaluation of success?

|  | **Enrichment plan** | **Behavioural goals** | **Evaluation of success** |
| --- | --- | --- | --- |
| **Yes** |  |  |  |
| **No** |  |  |  |
| **Somewhat** |  |  |  |
| **Notes** (If no, somewhat, please explain) |  |  |  |

**5.5.1** If so, is it made up of activities from all of the following five enrichment categories: food-based, cognitive, physical and structural, sensory, and social? Alternatively, please could you send us a copy of your enrichment plan?

|  | **Yes** | **Somewhat** | **No** | **Notes** (If somewhat, please list for which group(s) they don’t apply, which aren’t addressed and reason): |
| --- | --- | --- | --- | --- |
| **Food-based** |  |  |  |  |
| **Cognitive** |  |  |  |  |
| **Physical and Structural** |  |  |  |  |
| **Sensory** |  |  |  |  |
| **Social** |  |  |  |  |

**5.5.2** How frequently do you provide enrichment of the five categories (food-based, cognitive, physical and structural, sensory, and social) to all individuals? If you have multiple groups, please enter G1, G2, etc. in the relevant box.

|  | **Daily** | **Frequently (>1 week)** | **Occasionally (>1 month)** | **Rarely (<1 month)** | **Never** |
| --- | --- | --- | --- | --- | --- |
| **Food-based** |  |  |  |  |  |
| **Cognitive** |  |  |  |  |  |
| **Physical and Structural** |  |  |  |  |  |
| **Sensory** |  |  |  |  |  |
| **Social** |  |  |  |  |  |

**5.5.3** Do you always provide enrichment at the same time each day?

| **Yes** |  |
| --- | --- |
| **No** |  |

**5.5.4** Do you make provision for enrichment over night?

| **Yes** | **No** | **Notes** (If yes, please specify how): |
| --- | --- | --- |
|  |  |  |

**5.5.5** Does enrichment provision take into account social dynamics/structure?

| **Yes** | **No** | **Notes** (If no, please explain reason) |
| --- | --- | --- |
|  |  |  |

**5.5.6** Do you provide material for building nests, and what materials are provided and are they available during the day and at night?

|  | **Yes/No** | **Material** (Please specify materials here) |
| --- | --- | --- |
| **Daytime** |  |  |
| **Overnight** |  |  |

**5.5.7** Please provide a list of all enrichment items that you use. Alternatively, please can you send us a copy of your enrichment device inventory?

|  |
| --- |

**5.5.8** Do you perceive the following practices as routine husbandry, enrichment, or both?

|  | **Routine husbandry** | **Enrichment** |
| --- | --- | --- |
| **Scatter feed** (food items are thrown in various random directions throughout the enclosure |  |  |
| **Patchy distribution throughout enclosure** (food is distributed in piles throughout the enclosure) |  |  |
| **Puzzle Feeders** |  |  |
| **Self-feeders** |  |  |
| **Hiding food** |  |  |
| **Tool use tasks** |  |  |
| **Manipulable items** |  |  |
| **Browse** |  |  |
| **Changeable enclosure features** |  |  |
| **Animal Training** |  |  |
| **Nesting material** |  |  |
| **Other** (Please list here): |  |  |

**5.6** Please list the ways in which the individual apes can exert control over aspects of their lives. Please address the following aspects:

**1) Social** *(e.g., can choose to be alone or with other conspecifics, can escape, etc.)*

**2) Environmental** *(e.g., can be off-show at any times, can choose where to sleep at night, can use preferred enclosure features at all times such as moveable objects, malleable items like boomer balls, interactive mechanical and electronic devices, etc.)*

**3) Nutritional** *(e.g., can feed on a variety of food items, self-feeders, etc.)*

**4) Spatial/ temporal** *(e.g., can utilize preferred areas in the enclosure at all times, etc.)*

|  |
| --- |

- 1. Do you conduct voluntary positive reinforcement training (see definition section)?

| **Yes** |  |
| --- | --- |
| **No** |  |

**5.7.1** If so, please answer the questions below or send us a copy of all your ape training plans**.**

|  | **Notes** (please answer here) |
| --- | --- |
| **What are your target behaviours?** |  |
| **How often do you conduct training?** |  |
| **Is animal training guided by positive reinforcement?** |  |
| **Is positive reinforcement guided by an animal training plan?** |  |

- 1. Do you have facility design features or modifications that allow you to carry out operant condition training (e.g., training window, blood draw sleeves, etc.)? Please provide details below:

|  |
| --- |

1. **Healthcare**
   1. Do you have a Health Care Plan that includes regular health monitoring for all individuals and disease surveillance? If yes, provide details or leave blank otherwise.

|  |
| --- |

**6.2** How often do you monitor/test for each group (please enter G1, G2, etc. in the relevant box):

|  | **Daily** | **Weekly** | **Monthly** | **Up to 6 monthly** | **Up to yearly** | **Rarely** | **As required** | **Never** |
| --- | --- | --- | --- | --- | --- | --- | --- | --- |
| **Cardiovascular health** |  |  |  |  |  |  |  |  |
| - **Blood pressure** |  |  |  |  |  |  |  |  |
| - **Echocardiogram** |  |  |  |  |  |  |  |  |
| - **Electrocardiogram** |  |  |  |  |  |  |  |  |
| - **Intl. Primate Heart Project** |  |  |  |  |  |  |  |  |
| - **Great Ape Heart Project** |  |  |  |  |  |  |  |  |
| **Complete physical examination** |  |  |  |  |  |  |  |  |
| - **Ophthalmic** |  |  |  |  |  |  |  |  |
| - **Otic** |  |  |  |  |  |  |  |  |
| - **Dental** |  |  |  |  |  |  |  |  |
| - **Blood (parameters)** |  |  |  |  |  |  |  |  |
| - **Lymphatic** |  |  |  |  |  |  |  |  |
| - **Cardiovascular** |  |  |  |  |  |  |  |  |
| - **Respiratory** |  |  |  |  |  |  |  |  |
| - **Abdominal palpation** |  |  |  |  |  |  |  |  |
| - **Musculoskeletal** |  |  |  |  |  |  |  |  |
| - **Urogenital** |  |  |  |  |  |  |  |  |
| - **Neurologic** |  |  |  |  |  |  |  |  |
| **Body condition** (e.g., injuries, wounds, hair loss, etc.) |  |  |  |  |  |  |  |  |
| **Body condition Score** (note system being used) |  |  |  |  |  |  |  |  |
| **Weight** |  |  |  |  |  |  |  |  |
| **Faecal samples for evaluating pathogenic bacteria and parasites** |  |  |  |  |  |  |  |  |
| **Deworming** |  |  |  |  |  |  |  |  |
| **Urine** (e.g., Neopterin levels to monitor immune status) |  |  |  |  |  |  |  |  |
| **Activity levels** |  |  |  |  |  |  |  |  |
| **Vaccination** |  |  |  |  |  |  |  |  |
| **Joint/ muscular degeneration** |  |  |  |  |  |  |  |  |
| **Needs of geriatric individuals** |  |  |  |  |  |  |  |  |
| **Needs of those with chronic conditions** |  |  |  |  |  |  |  |  |
| **Other** (Please list here): |  |  |  |  |  |  |  |  |

1. **Staff Training**
   1. How often do your ape keepers attend species-specific continued professional development opportunities (e.g., GAWg workshops, BIAZA certified workshops; ABWAK workshops)?

|  |
| --- |

- 1. Do you fund some or all attendance at CPD workshops?

| **Some** |  |
| --- | --- |
| **All** |  |

Zoo Facilities & Management Survey

- Part II -

**This is survey part II, which contains mostly green-orange highlighted questions that we will answer together and that can be answered by me alone during my visit. If possible, please complete the blue-highlighted boxes in advance.**

**Please complete one survey document per species.**

**Please note, if you hold multiple groups of the same species in a single facility but with no shared space this will be considered multiple facilities and you should complete a different questionnaire for each facility.**

**If you hold all groups of the same species that rotate through the same enclosure spaces this would be treated as one facility, so one questionnaire would be answered but please code the groups G1, G2, G3 etc.**

**Interpretation of terms used**

The following terms as defined here are used in this zoo facility and management survey.

- **Indoor enclosure:**  publicly-viewable inside area of an exhibit.
- **Outdoor enclosure:** publicly-viewable outside area of an exhibit.
- **Off-show areas:** Unless specified this term refers to the primary off-show areas that apes regularly use, rather than secondary off-show areas which are rooms used for specific purposes (such as quarantine, introductions, hand-rearing, medical treatment, specialized research observation, etc.).
- **Enclosure barrier:** The physical barrier that contains the animals in the enclosure.
- **Visual barrier:** Visual barrier that enables animals to hide from public view and/or be out of sight of conspecifics, but may be made from flexible material.
- **Three-dimensional off-ground locomotor structures:** Vertical, horizontal and angled pathways with cross-connections between them at variable height levels.
- **Positive reinforcement:** The introduction of a desirable or pleasant stimulus (i.e., a reward) after a behaviour. The desirable stimulus reinforces the behaviour, with the intention of making it more likely that the behaviour will occur again.

1. **Enclosure**

**Note: Primary off-show areas are areas off-show to the public that apes access regularly (e.g., sleeping dens). Secondary off-show areas are areas off-show to the public that are only used in exceptional circumstances (e.g., for introductions).**

- 1. Please provide the following information:
- The ground area of space that your species have access to and the maximum height attainable (in outdoor enclosures this will be defined either by a roof/netting or the maximum height of climbing equipment)?
- Number of access points to each room?
- What is the maximum number of individuals that are housed in each room regularly?
- For how many hours in a 24 hour cycle does the maximum number of individuals have access to each room?

**If you have multiple groups, please enter for each group: G1: “*Max No. of apes*”, G1: “*No. of hours*“.**

|  | **Indoor** | | | | | **Outdoor** | | | | | **Primary off-show** | | | | |
| --- | --- | --- | --- | --- | --- | --- | --- | --- | --- | --- | --- | --- | --- | --- | --- |
| **Rooms** | **Area (m^2^)** | **Height (m)** | **No. access points** | **Max No. of apes** | **No. of hours** | **Area (m^2^)** | **Height (m)** | **No. access points** | **Max No. of apes** | **No. of hours** | **Area (m^2^)** | **Height (m)** | **No. access points** | **Max No. of apes** | **No. of hours** |
| **1** |  |  |  |  |  |  |  |  |  |  |  |  |  |  |  |
| **2** |  |  |  |  |  |  |  |  |  |  |  |  |  |  |  |
| **3** |  |  |  |  |  |  |  |  |  |  |  |  |  |  |  |
| **4** |  |  |  |  |  |  |  |  |  |  |  |  |  |  |  |
| **5** |  |  |  |  |  |  |  |  |  |  |  |  |  |  |  |
| **6** |  |  |  |  |  |  |  |  |  |  |  |  |  |  |  |
| **7** |  |  |  |  |  |  |  |  |  |  |  |  |  |  |  |
| **8** |  |  |  |  |  |  |  |  |  |  |  |  |  |  |  |
| **9** |  |  |  |  |  |  |  |  |  |  |  |  |  |  |  |
| **10** |  |  |  |  |  |  |  |  |  |  |  |  |  |  |  |

- 1. What is the maximum height that your apes use on a daily basis (which may differ to the maximum height available)?

| **Indoor (m)** | **Outdoor (m)** | **Primary off-show (m)** |
| --- | --- | --- |
|  |  |  |

- - 1. Please tick which specific age groups use the maximum height of your enclosures on a daily basis.

**Note: Age guidelines are based on data from the wild.**

|  | **Indoor** | **Outdoor** | **Primary off-show** |
| --- | --- | --- | --- |
| **Elderly adults** (Chimps: ~33+; Bonobos: ~32+; Gorillas: ~35+; Orangutans: ~35+) |  |  |  |
| **Adults** (Chimps: 15+; Bonobos: 15+; Gorillas: 11+; Orangutans: 18+) |  |  |  |
| **Juveniles** (Chimps: 5-8; Bonobos: 4-7; Gorillas: 4-7.5; Orangutans: 4-8) |  |  |  |
| **Infants** (Chimps: <5; Bonobos: <4; Gorillas: <4; Orangutans: <4) |  |  |  |

- 1. Do your individual animals have continual access to drinking water?

| **Yes** | **No** | **Notes** (If no, please explain) |
| --- | --- | --- |
|  |  |  |

- 1. When outside, do your animals have access to shelter for all individuals to use at the same time? *(A shelter is defined as* *an outdoor structure that apes can use to be protected from extreme weather. This can be multiple outside shelters in different areas or only one shelter).*

| **Yes** | **No** | **Notes** (If no, please explain) |
| --- | --- | --- |
|  |  |  |

- 1. Please tick the height levels for which your enclosures provide three-dimensional off-ground locomotor structures? *(A locomotor structure refers to anything the animal can use for movement. Height levels are defined as low: 1m above ground; medium: space between low-high; high: 1m below indoor ceiling or below highest outdoor climbing structure).*

|  | **Indoor** | | | **Outdoor** | | | **Primary off-show** | | |
| --- | --- | --- | --- | --- | --- | --- | --- | --- | --- |
| **Rooms** | **Low** | **Medium** | **High** | **Low** | **Medium** | **High** | **Low** | **Medium** | **High** |
| **1** |  |  |  |  |  |  |  |  |  |
| **2** |  |  |  |  |  |  |  |  |  |
| **3** |  |  |  |  |  |  |  |  |  |
| **4** |  |  |  |  |  |  |  |  |  |
| **5** |  |  |  |  |  |  |  |  |  |
| **6** |  |  |  |  |  |  |  |  |  |

- 1. Please describe the rigid, flexible and other weight-bearing structures (as defined below) that are present in all similar spaces combined (e.g. indoor or outdoor)? *Rigidity is defined as the behaviour of the support under the animals weight.*
- *Rigid supports (e.g., poles, logs, platforms, metal bars, steel mesh) that are vertical, horizontal, angled*
- *Flexible supports (e.g., single ropes, cargo nets, hammocks, webbing) that are vertical, horizontal, angled*
- *Other objects that can be used for weight bearing (e.g., barrels, tyres, etc.)*

Please list:

1. Number **(No.)** of supports
2. Type score **(TS)**: (1) all same type of support; (2) 2-4 types; or (3) 4+ types
3. Orientation score **(OS)**: (1) all same; (2) mostly 2 types; (3) all three types are equally represented, where the types are horizontal (± 20° from true horizontal), vertical (± 20° from true vertical) or angled (all other orientations).

|  | **Indoor** | | | **Outdoor** | | | **Primary off-show** | | |
| --- | --- | --- | --- | --- | --- | --- | --- | --- | --- |
|  | **No.** | **TS** | **OS** | **No.** | **TS** | **OS** | **No.** | **TS** | **OS** |
| **Rigid** |  |  |  |  |  |  |  |  |  |
| **Flexible** |  |  |  |  |  |  |  |  |  |
| **Other** |  |  |  |  |  |  |  |  |  |

- 1. Can all individuals access all structures equally without any animals being excluded (e.g., some individuals can monopolize, etc.)?

| **Yes** | **No** | **Somewhat** | **Notes** (If no, somewhat, please explain) |
| --- | --- | --- | --- |
|  |  |  |  |

- 1. Which of these barriers are used in your enclosure?

| **Enclosure barrier** | **Indoor** | **Outdoor** | **Off-show** (incl. primary and secondary off show areas) |
| --- | --- | --- | --- |
| **Dry moat** |  |  |  |
| **Wet moat (accessible to the apes)** |  |  |  |
| **Electric wire/fence as primary barrier** |  |  |  |
| **Electric wire/fence as secondary barrier** |  |  |  |
| **Steel bars** |  |  |  |
| **Concrete wall** |  |  |  |
| **Glass wall** |  |  |  |
| **Glass windows** |  |  |  |
| **Other** (please list here) |  |  |  |

**2.12** Is there a wet moat that your apes can access? If so, complete below:

|  | **Yes** | **No** |
| --- | --- | --- |
| **Is there a gradual gradient on the apes’ side?** |  |  |
| **Are there supports to help apes getting out?** |  |  |

1. **Social grouping**

**4.5** If there are multiple groups, do they have the opportunity to interact with each other? Please fill in Yes when applicable.

|  | **Group 1** ↔ **Group 2** | **Group 1** ↔ **Group 3** | **Group 2** ↔ **Group 3** |
| --- | --- | --- | --- |
| **Physical** |  |  |  |
| **Visual** |  |  |  |
| **Auditory** |  |  |  |
| **Olfactory** |  |  |  |

1. **Feeding and Nutrition**
   1. Does the zoo have a feeding and nutrition plan that accounts for each individuals’ age and nutritional requirements and includes regular monitoring of intake?

|  | **Age** | **Nutrition** | **Monitoring** |
| --- | --- | --- | --- |
| **Yes** |  |  |  |
| **No** |  |  |  |
| **Somewhat** |  |  |  |

- 1. Which of these feeding methods do you use? If you have multiple groups, list G1, G2, etc. in the relevant box.

|  | **Daily** | **Frequently (>1 week)** | **Occasionally (>1 month)** | **Rarely (<1 month)** |
| --- | --- | --- | --- | --- |
| **Scatter feed** (food items are thrown in various random directions throughout the enclosure) |  |  |  |  |
| **Patchy distribution throughout enclosure** (food is distributed in piles throughout the enclosure. |  |  |  |  |
| **Hand-fed** |  |  |  |  |
| **Controlled feeding** (separating individuals out) |  |  |  |  |
| **Self-feeder** |  |  |  |  |
| **Arboreal feeding (e.g. through mesh)** |  |  |  |  |
| **Hidden arboreally** |  |  |  |  |
| **Hidden in vegetation** |  |  |  |  |
| **Hidden in substrate** (e.g., sand, deep bedding of bark, soil etc.) |  |  |  |  |
| **Other** (Please list here): |  |  |  |  |

- 1. How many times do you feed your apes per day?

|  |
| --- |

- 1. Do you always feed them at the same time each day?

| **Yes** |  |
| --- | --- |
| **No** |  |

**8.5** Do feeding methods take account of the group’s dominance hierarchy to give all group members the opportunity to access preferred foods and/or a balanced diet?

|  | **Yes** | **No** | **Notes** (If yes, specify methods here): |
| --- | --- | --- | --- |
| **Preferred foods** |  |  |  |
| **Balanced diet** |  |  |  |
