# Supplementary material for: Developing and validating a holistic welfare assessment tool for zoo-housed great apes: Integrating resource-based measures with behavioural ecology insights
Source: PLoS One. 2026 Jan 30;21(1):e0340094. doi: 10.1371/journal.pone.0340094 (PMC12858012; doi:10.1371/journal.pone.0340094)
Supplement: S2 File — (DOCX) [file pone.0340094.s002.docx]

**Supplementary Results**

**Containment barriers**

Wild populations are only contained by features such as rivers, neighbouring chimpanzee troops and in some cases human settlements. The types of barrier used for surrounding the primary living area for captive chimpanzees is dependent upon several factors including terrain, climate, and construction and maintenance costs. The main types of barriers include walls, wet moats, dry moats, wire mesh fencing, and electric fencing. However, water moats can be problematic as chimps are poor swimmers and can drown after jumping or falling into moats (Adang et al, 1987; McDonald, 1994). Such accidents have led to the addition of underwater supports or sloping sides to help chimps climb out, or an additional barrier (e.g., electric wires) to prevent them from wading too far into the water (McDonald, 1994). There is of course little evidence from the wild that can be applied for this component of husbandry and such cases are not well documented (e.g., Angus, 1971; Farmer, 2002). Some authors suggest that exhibits should be designed to limit the ability of chimpanzees to use trees, structures, or sloping hills to extend jumping heights as they are capable of jumping a horizontal distance of up to 6 m (Coe et al., 2001), however this clearly conflicts with the need to utilise as much space as possible. Overall, there is only limited data to determine which barriers support positive welfare and which are preferred by chimpanzees (Ross & Lukas, 2006), although chimpanzees have been observed to use mesh panels significantly more often than spending time adjacent to public glass barriers and solid concrete walls. Concrete can transmit warmth from internal rooms or absorb it from the sun, providing warm spots for chimps outdoors. Glass is clearly central to the apes being on display but can contribute to poor welfare if the chimpanzees cannot get out of sight of visitors.

Concrete walls, glass windows/glass walls, and thick mesh were the main components of enclosure barriers used between visitors/staff and animals in this study. Concrete was used in six indoor, five outdoor and five off-show areas; glass was used in six indoor, five outdoor and two off-show and thick mesh was used in two indoor, three outdoor and six off-show enclosures. Four zoos also had steel bars. No zoo had a dry moat barrier outdoors but three had a deep-water moat outdoors that was accessible to the chimpanzees and/or a shallow wet moat indoors. All wet moats had a gradual gradient, but only one also had supports to help the chimpanzees get out of the water easily. Five zoos used electrical fences as a secondary barrier outdoors and one also used an electrical fence/wire as a secondary barrier indoors. Overall, the low usages of mesh for indoor enclosures indicates that enclosure structure at most zoos constraints the chimps’ potential to utilise the entire volume of their indoor enclosure, as mesh is a key component for creating arboreal locomotion opportunities (via attachments points for ropes as a climbable structure). It can allow enclosures with a comparatively small ground area but reasonable height to provide higher welfare by facilitating access to the maximum enclosure volume, increasing opportunities for natural climbing locomotion and social separation. This holistic picture is clearly informative for predicting actual welfare during times of reduced enclosure access.

**Environmental conditions**

Wild chimpanzee low-land rainforests are characterized by little change in temperature from season to season (ranging from 18°C- 26°C) and high humidity (averaging 77-85%) (Boesch & Boesch-Achermann, 2000; Takemoto, 2004). In contrast to more extreme habitats like arid areas, rainforest chimpanzees experience overall less seasonal stress (indicated by cortisol levels) but do appear to face dehydration pressure (indicated by creatinine levels), if relative humidity is too low (<40%; Wessling et al., 2018). Little research has been carried out on optimal levels of humidity for chimpanzee indoor enclosures. Clutton-Brock (1979) recommended between 30-70% to match levels at Gombe forest during the dry season. However more research is needed to quantify the extent of thermoregulatory stress that wild chimpanzees experience in their natural environments before zoos match the environmental conditions of captive habitats to wild ones. Unfortunately, a direct question regarding humidity was erroneously omitted from the final survey. However, the observer took informal notes during data collection, which revealed that although some of the zoos had a humidity measurement device that they assessed sporadically, none of them routinely recorded humidity.

Wild chimpanzees are also exposed regularly to natural sunlight and since they require vitamin D, it is essential that captive chimps have adequate exposure to sunlight (Holick, 1994). Ensuring sufficient Vitamin D is particularly important when chimpanzees are housed predominantly indoors or are not exposed to sunlight during the winter in northern climates. In seasons where UV levels are naturally low, circulating vitamin D levels have been reported to be low in zoo-housed chimpanzees, an aspect linked to cardiovascular disease in people (Strong et al 2019). Unfortunately, there is a lack of empirical evidence for required dosage of natural vitamin D exposure or supplementation (and we did not query supplementation in the survey).

The soundscape, including both frequency and sound pressure levels, also appears to be important in chimpanzee welfare. Research shows that chimpanzees are particularly attuned to sounds between ~1 kHz and 8 kHz (Kojima, 1990). They are less sensitive to lower frequencies (below 250 Hz and 2- to 4 kHz tones) compared to humans, but more sensitive to higher frequencies (Kojima, 1990). Some data suggest that noise generated by visitor groups can reach sound pressure levels outside of the recommended limits for human well-being (>70 dBA; WHO 1999) and thus may lead to negative welfare in zoo animals. For example, circular enclosures, such as islands, have a greater area for public access. Popular species like chimpanzees attract larger visitor crowds, leading to elevated noise levels that makes chimpanzees more vigilant and move around more (63.5 - 66.5 ± 4.75 – 6.25 dBA; Quadros et al., 2014). High noise levels have been shown to also decrease other behaviours, such as foraging, grooming, and play (Wood, 1998).

Overall, this is an area that is little understood and undermeasured by zoos. Temperature was measured at six zoos and ranged from 15 - 28°C for indoor on-show exhibits and 12 - 25°C for the off-show areas Table SI3.1); the lower off-show temperatures appears relatively cold, but there is no evidence to date regarding the lowest temperature that chimpanzees are comfortable at, and how this varies if they have the option to move to warmer spaces when needed. Seven zoos did not provide artificial UV; the zoo that did measured 22 mW/cm^2^ in their latest light intensity reading (although we did not query how far from the light source the reading was taken). Four zoos had UV permeable windows indoors. None of the zoos sampled humidity and six didn’t provide artificial UV indoors, despite limited access outdoors during the winter (note that the survey did not ask about Vitamin D supplementation). All zoos reported that it was possible to hear visitor noise, staff vehicles, internal machinery, metal slides and doors and the carpark in both indoor and outdoor spaces. Most had mitigation strategies in place that reduced the apes’ exposure to undesirable noise (e.g., sound-proof/ thick glass; signage; keepers working as quietly as possible; outdoor vegetation, waterfalls and visitor pathways designed to maintain distance from the apes). Similarly, five zoos with an indoor on-show exhibit and all zoos with an outdoor area reduced the visual impact of their visitors to some extent using husbandry management (e.g., off-show access, low light levels on visitor corridors, provision of hessian sacks or browse) and built-in enclosure design, such as visual barriers, wet moat, topography, vegetation and shelters. One mitigated for both by its isolated location and visitors viewing via organised boat trips rather than pathways. Overall, there is currently a limited evidence base to establish the environmental conditions that promote good welfare and where the boundary with poor welfare lies. Clearly, these are important components of the holistic experience of the chimpanzees and need to be incorporated into future welfare considerations and assessment.

**Table S2.1. Environmental conditions.**

| **Zoo** | **1** | **2** | **3** | **4** | **5** | **6** | **8** |
| --- | --- | --- | --- | --- | --- | --- | --- |
| **Artificial UV provision** | N | N | Y | N | N | N | N |
| **Frequency of UV readings** | n/a | n/a | Week | n/a | n/a | n/a | n/a |
| **UV permeable ceilings and/or windows** | Y | N | N | Y | Y | Y | N |
| **Indoor temperature** | 18-25° | 24° | 24° | 15-28° | 18-20° | 22° | n/a |
| **Off-show temperature** | 22-25° | 22° | 24° | unk | 22-24° | N/A° | 18-19° |
| **Reduce noise exposure indoor** | Y | Y | Y | Y | Y | Y | n/a |
| **Reduce noise exposure outdoor** | Y | Y | Y | Y | N | Y | Y |
| **Reduce noise exposure off-show** | N | Y | Y | Y | N | n/a | N |
| **Reduce visual impact of visitors indoor** | Y | Y | Y | Y | Y | N | n/a |
| **Reduce visual impact of visitors outdoor** | Y | Y | Y | Y | Y | Y | Y |

Colours indicate good (pale blue), moderate (mid blue – none present) or lower (dark blue) welfare, based on the evidence presented in the discussion. Parameters are not colour coded if the evidence for welfare is not yet established. Week: weekly, unk: unknown, n/a: not applicable.

**Environmental Enrichment**

Wild chimpanzees face many daily challenges in their complex environment, and these must be met using their enhanced cognitive skills (e.g., Meehan & Mench, 2007; Shettleworth, 2010), such as spatial orientation, long-term memory, and long-term planning of movements. These skills enable them to take efficient routes to their destinations (e.g., Boesch & Boesch, 1984; Bates & Byrne, 2009; Normand & Boesch, 2009; Ban et al., 2014). They spend a high proportion of time foraging, exploring their environment, and interacting with conspecifics (e.g., Goodall, 1986; Amsler, 2009; Brosnan & Hopper, 2013), all of which are cognitively demanding. Moreover, wild chimpanzees are extractive foragers who make and use many different tools to obtain otherwise inaccessible foods (Whiten, 2011).

Captive chimps, however, live in smaller and simpler habitats, and are rarely required to make use of their cognitive skills when looking for food; a key activity for their physical and mental stimulation (e.g., Ban & Normand, 2019). Environmental enrichment has traditionally been split into five main categories (Bloomsmith et al., 1991). These are: (1) food-based; (2) occupational (including psychological ‘puzzles’ and physical exercise); (3) structural (e.g., enclosure design); (4) sensory (e.g., visual, auditory); and (5) social. As described above, the presentation of food is a critical form of enrichment and can help increase foraging time and decrease aggression and abnormal behaviour (Bloomsmith et al., 1988). ‘Substrate feeders’ have been shown to increase the duration of foraging behaviours and overall activity in captive chimpanzees (Lambeth & Bloomsmith, 1994; Baker, 1997). Food-based devices such as ‘stuffed boxes’ and ‘probe feeders’ elicit manipulation, such as individuals ‘dipping’ their fingers or a tool into a chamber to remove sticky food items (Maiki et al., 1989; Celli et al., 2003, Zaragoza et al., 2011). Highly complex and/or unpredictable cognitive tasks have been reported as enriching for zoo living chimpanzees (e.g., Bloomsmith et al., 2000; Clark & Smith, 2013; Brooks et al., 2021). Similarly, providing novel and manipulable items or rotating different devices regularly (Wallace, 1988), increases object manipulation, natural behaviours, permits control, and reduces inactivity and stereotyped/self-directed abnormal behaviours among captive chimpanzees (e.g., Paquette & Prescott, 1988; Bayne, 1989; Shefferly et al., 1993; Sambrook & Buchanan-Smith, 1997; Videan et al., 2005). Other research demonstrates that the provision of physical and structural enrichment increases activity, such as exploring activities (Zaragoza et al., 2011) or challenging chimpanzees to use tools (e.g., Celli et al., 2003), play with unpredictable ropes (Chamove, 1989) and to travel longer distances to obtain more‐preferred food rewards (Hopper et al., 2016). However, evidence shows that food-based devices are commonly monopolized by dominant individuals and can lead to frustrated competition and thus it is important to deliver enrichment that successfully considers the social dynamics/ structures of the chimpanzee group (e.g., Bloomstrand et al., 1986; Bloomsmith et al., 1988; Maiki et al., 1989; Celli et al., 2003).

A final activity that has relevance to enrichment and management is operant conditioning or positive reinforcement training (PRT), which offers benefits for both for chimpanzees and staff (Bloomsmith et al., 1999; Lambeth et al., 2006; Laule & Whittaker, 2007; Pomeratz & Terkel, 2009; Perlman et al., 2010). Research shows that these activities provide chimpanzees with important elements of choice and control, contributing to their psychological well-being (Laule & Whittaker 2001). Likewise, the voluntary cooperation that results from training programmes reduces the need for physical interventions for medical procedures, and the accompanying risks associated with those events (Bloomsmith 1992; Reinhardt et al., 1995).

While no zoo had a written environmental enrichment plan (Table SI3.2) with defined behavioural goals and evaluation measures, three did plan environmental enrichment to some extent, with some consideration of behavioural goals and evaluation of success. The remaining zoos delivered environmental enrichment on a more *ad hoc* basis. For these, evaluation of success was either included in daily welfare assessment by the keepers or was *ad hoc*. Overall, seven zoos provided food-based enrichment daily; six provided cognitive enrichment daily, and two stated they enriched their chimps socially every day. Physical and structural enrichment occurred the least frequently. The most enrichment was provision of all five types on a daily basis (see Table SI3.3 for the qualitative list of enrichment items provided at the zoos). Six zoos varied the time of day that they provided enrichment and five provided some form of overnight enrichment sometimes or always. Six zoos considered the social dynamics/structures of their chimpanzee group when providing enrichment to minimise competition over enrichment devices and risk of aggression. The zoo that did not do so considered it unnecessary as their group was small.

**Table S2.2**. **Enrichment planning, management, and provision by the zoos.**

| **Zoo** | **1** | **2** | **3** | **4** | **5** | **6** | **8** |
| --- | --- | --- | --- | --- | --- | --- | --- |
| **Enrichment Plan that^a^:** | N | N | N | S | S | S | N |
| addresses behavioural goals | N | N | N | Y | Y | S | N |
| includes evaluation of success | S | N | N | Y | Y | S | N |
| includes food based enrichment | n/a | n/a | n/a | Y | Y | Y | n/a |
| includes cognitive enrichment | n/a | n/a | n/a | Y | Y | Y | n/a |
| includes physical and structural enrichment | n/a | n/a | n/a | Y | Y | S | n/a |
| includes sensory enrichment | n/a | n/a | n/a | Y | Y | S | n/a |
| includes social enrichment | n/a | n/a | n/a | Y | Y | S | n/a |
| **Frequency of enrichment^b^:** |  |  |  |  |  |  |  |
| Food based | daily | daily | daily | daily | daily | daily | daily |
| cognitive | daily | daily | daily | daily | daily | daily | >1 week |
| physical and structural | daily | <1 month | >1 week | >1 month | <1 month | >1 month | >1 month |
| sensory | daily | <1 month | >1 week | >1 week | >1 week | >1 month | <1 month |
| social | daily | never | >1 week | daily | >1 week | <1 month | never |
| **Timing/ nature of enrichment^a^:** |  |  |  |  |  |  |  |
| provided at same time each day | N | N | N | N | Y | N | N |
| provided overnight | Y | Y | Y | N | Y | N | Y |
| accounts for social dynamics | Y | Y | Y | Y | Y | Y | n/a |
| **Nesting material^c^:** |  |  |  |  |  |  |  |
| Types of nesting material provided - day | OP,T,W | OP,W | B,CP,T,W | OP,T,W | T,W | B,CP,T,W | OP |
| Types of nesting material provided - night | T,W | OP,W | B,CP,T,W | OP,T,W | B,T,W | B,CP,T,W | OP |

Y: Yes; N: No; S: Somewhat.

^a^For questions relating to the enrichment plan and timing/nature of enrichment, the text in the cells is the zoos actual response to the fixed outcome questions, but the colour coding takes into account their explanations in the free text questions. Thus, for questions relating to the enrichment plan, pale green means no full written plan but some unwritten planning or written planning for some components, and mid blue means ad hoc provision of enrichment and informal consideration of behavioural goals and evaluation, even though the zoos responded that they had no written plan. Parameters are not colour coded if the evidence for welfare is not yet established.

^b^The question regarding frequency of enrichment allowed 5 responses, so colours indicate very good (pale blue), good (pale green) moderate (mid blue), low (dark green) and very low (dark blue) aspects of welfare, based on the evidence presented in the discussion.

^c^For types of nesting material: B: browse, CP: cardboard/paper, OP: other plant material (e.g. straw, grass, hay, haylage), T: textiles (e.g. sacks, old clothes/towels, blankets, bed sheets, duvets, W: wood-based materials (e.g. wood wool, wood shavings).

**Table S2.3: Qualitative responses regarding enrichment items provided**

| **Zoo** | **Enrichment provided** |
| --- | --- |
| **1** | webbing, cargo nets, fire hose parcels, tyres, sticks, wood-wool, sacks, built-in holes in wall |
| **2** | paper mache items, plastic bottle, cardboard boxes, paper feed sacks, hessian sacks, hose lengths, cloths, large dog toys, scent sprays, nontoxic paints, crayons, camel hair, straw and hay bedding, bark chips, browse, leaf piles, balls, kongs, puzzle feeders, hosepipe showers, ice blocks, mobile furnishings, furniture changes, condiments, disco lights, radio, tv, novel food items |
| **3** | pipes of mashed potato or porridge, firehose lengths, cardboard boxes with food, toilet rolls with food, firehose cubes, firehose twists, browse, herbs, plastic bottles, plastic containers, frozen foods, blankets, sheets, clothing, puzzle feeders, termite mound, substrate piles, novel items, perfumes, spices, new furniture, rotating pvc piping, sacks, smears of yoghurt on climbing frames, fruit kebabs, whole coconuts and pumpkins, Christmas trees, kerplunk tubes |
| **4** | boomer balls, barrels, ropes, strapping, logs, branches, kongs, frozen bottles, ice blocks, herbs, cardboards boxes, sheets, duvets, play balls, football, cargo nets, puzzle feeder, tubes, rope boards, spinning bottle feeders, spinning ball feeders, hose feeders, perfumes |
| **5** | alternative food items, astro turf feeder, boomer ball, bottle, box, browse, cage ball feeder, cage feeder, egg carton, enclosure alteration, extra drink, firehose sausage/cubes, firehose segments, fishing, flour & seed mix, food parcel, food paste, frisbee tower, herbs, hessian sack, hidden food, ice cubes, individual frisbees, jelly, juice, keyboard, kong, live insects, log feeder, mirror, novelty food, nuts, painting, paper & plastic cubs, paper mâché, paper plaits, paper sack, pineapple top, pipe feeder, plant pots, plastic bottle, plastic bucket, plastic drain feeder, pvc pipe, radio, scatter feed, scents, spinning bottle feeder, strap feeder, tennis balls, toilet roll, video, wadger, water, wooden block feeder, whole vegetables |
| **6** | plant pot feeder, basket feeder, frozen firehose strips, hanging barrels, hanging feeders, ice blocks, kerplunk tubes, shopping basket feeders, suspended net feeders, bubbles, duvets, paper chains, xmas presents, wolf howling sound device, brown paper reels, carpet tubes, |
| **8** | feedpod, puzzle feeders, kongs, hanging food boxes, hanging log with holes, ground log with holes, containers |

Only one zoo didn’t carry out positive reinforcement training (PRT) with their chimpanzees (Table SI3.4); three zoos conducted training daily and three weekly. In three zoos training was guided by a formal training plan, one zoo used a plan only loosely, and two zoos had no plan. Five had enclosure design features that supported training sessions. Three zoos targeted more than four different actions/ behaviours during PRT training.

**Table S2.4. Structure and contents of positive reinforcement training.**

| **Zoo** | **1** | **2** | **3** | **4** | **5** | **6** | **8** |
| --- | --- | --- | --- | --- | --- | --- | --- |
| **Structure of training***^a^***:** |  |  |  |  |  |  |  |
| Conduct voluntary positive reinforcement training | Y | Y | Y | Y | Y | Y | N |
| Frequency of training | daily | daily | >1 week | > 1 week | daily | > 1 week | n/a |
| Animal training guided by positive reinforcement | Y | S | Y | Y | Y | Y | n/a |
| Positive reinforcement guided by an Animal Training Plan | N | N | S | Y | Y | Y | n/a |
| Facility features to support training | N | N | Y | Y | Y | Y | Y |
| **Training contents***^b^***:** |  |  |  |  |  |  |  |
| Body part presentation |  |  |  |  |  |  |  |
| Scales |  |  |  |  |  |  |  |
| Moving around enclosures |  |  |  |  |  |  |  |
| Separation |  |  |  |  |  |  |  |
| Xray/ ECG/ ultrasound |  |  |  |  |  |  |  |
| Urine sample |  |  |  |  |  |  |  |
| Injections/ blood draw |  |  |  |  |  |  |  |
| Administering medications/ nebuliser |  |  |  |  |  |  |  |
| Emergency recall |  |  |  |  |  |  |  |
| Proximity to keepers |  |  |  |  |  |  |  |
| Ear temp/ nose swab |  |  |  |  |  |  |  |
| Station |  |  |  |  |  |  |  |
| Nail trimming |  |  |  |  |  |  |  |
| Open mouth |  |  |  |  |  |  |  |
| Wound management |  |  |  |  |  |  |  |

*^a^For structure, the colours indicate good (pale blue) moderate (mid blue), low (dark blue) aspects of welfare, based on the evidence presented in the discussion. N/a: not applicable. ?: no response to the question. Parameters are not colour coded if the evidence for welfare is not yet established.*

*^b^For training content grey indicates training was undertaken.*

**Welfare management**

There is currently no evidence for chimpanzees on how often and at what time of day welfare assessments should be conducted. Nevertheless, it is recognized that welfare is a state that varies across time/seasons/places and thus welfare should be assessed several times throughout a year and at varying times of day (e.g., Whitham & Wielebnowski, 2009). Several studies have reported the need to combine multiple positive and negative indicators of welfare to provide a reasonable insight into the animal’s welfare state (e.g., McCormick et al., 2012; Carlstead et al., 2013; Clegg et al., 2015; Sherwen et al., 2018; Wolfensohn et al., 2018). Wolfensohn et al., (2018) also argued for the presence of independent observers to ensure objectivity. It is also vital that the welfare indicators used have been assessed for reliability and validity to ensure they can be consistently applied and that there is good supporting evidence for their use as indicators (Yon et al. 2019); this remains a major challenge in the field.

In this study six zoos assessed welfare (Table SI3.5). Five did so on a daily basis; three specified the number of times per day that assessment was carried out (and the remaining two reported that they assessed welfare on an ad hoc basis). One zoo responded that they only assessed welfare annually. The diversity in these results likely indicates lack of clarity as to whether the question was referring to standardised welfare assessment protocols or any welfare monitoring/ assessment activities. No zoo had a set duration for which each animal was observed. Welfare assessments were guided by an assessment tool at three zoos. Note that the content of welfare assessment tools and policies were not assessed here, and the survey also did not assess whether the tools had been assessed for reliability. No zoos involved independent (e.g. external to the zoo) observers in welfare assessments, but two used multiple teams (e.g. keepers, vets, nutrition, boat drivers, and section leads from other sections). One zoo stated that keepers were the most appropriate people to assess welfare as they know the animals best and the chimps behave differently in front of other people. Six zoos said that multiple people/groups were responsible for the planning, implementing and monitoring of welfare, including keepers, team leaders, research and welfare teams, curators and senior managers. While six listed keepers as being responsible for implementing and measuring welfare measures, only four listed keepers as being involved in planning welfare. Only one zoo had a single person (Team lead) responsible for planning, implementing and monitoring welfare. Welfare is complex and managing welfare for every species in a zoo is an enormous task that requires a systematic approach, teamwork and access to the latest science to accomplish good welfare outputs for the animals in care. Many zoos have limited resources (i.e., staff, budget, time) and thus assessing the welfare of all species/individuals they care for is an enormous challenge, particularly when only a small number of people are available.

**Table S2.5. Welfare planning, management and provision by the zoos.**

| **Zoo** | **1** | **2** | **3** | **4** | **5** | **6** | **8** |
| --- | --- | --- | --- | --- | --- | --- | --- |
| **Undertake welfare assessment** | Y | N | Y | Y | Y | Y | Y |
| Frequency | daily | - | 2 pd | 6 pd | 3 pd | annual | daily |
| Time of day | ad hoc | - | am/pm | am, lunch, pm | am, lunch, pm | ad hoc | ad hoc |
| Duration per animal | no set duration | - | no set duration | no set duration | no set duration | no set duration | no set duration |
| Guided by assessment tool | N | - | N | Y | Y | Y | N |
| Includes independent observers | N | - | N | Y | N | Y | N |
| **Staff responsible for***^a^***:** |  |  |  |  |  |  |  |
| planning welfare | K, TL, C | K, TL, SM | TL | R, W, SM | C, SM | K, TL, C, SM | K, TL, R, SM |
| implementing welfare | ALL | K, TL, SM | TL | K, TL | K, SM | K, TL, C | K, TL, SM |
| monitoring welfare | K, TL, R, C | K, TL | TL | K, TL | K, SM | K, TL, C | K, R |
| Welfare assessed during acquisition, transfer, transition policy | S | Y | Y | Y | Y | Y | Y |
| Keepers can attend CPD | Y | Y | Y | Y | Y | Y | Y |
| Funding provided for CPD | y | y | y | y | y | s | y |
|  |  |  |  |  |  |  |  |
| **Written welfare plan that addresses***^b^***:** | Y | N | N | Y | Y | Y | N |
| Behavioral measures | P, N, M | N | N | P, N, M | P, N | P, N | N |
| Physical measures | P, N, M | N | N | P, N, M | P, N | P, N | N |
| Physiological measures | P, N, M | N | N | P, N, M | P, N | N | N |
| Psychological measures | P, N, M | N | N | P, N, M | P, N | N | N |

Y: yes, N: no, S: somewhat.

^a^For questions regarding staff responsible for welfare K: keepers, TL: team leader, R: research team, C: curator, SM: senior managers; W: welfare team.

^b^For written welfare plan questions P: positive welfare indicators, N: negative welfare indicators, M: monitoring. Colours indicate good (pale blue) moderate (mid blue), low (dark blue) aspects of welfare, based on the evidence presented in the discussion. Colour coding is only applied where there is evidence to support classifications.

Four zoos had a written Welfare Plan; three of which included positive and negative welfare states for behavioural, physical, physiological, and psychological components, while one focused on behavioural and physical components only. Only two incorporated mitigation strategies for welfare issues into their Plans. Three submitted an electronic copy of their welfare plan, which showed that they recorded 16-34 indicators of positive and negative welfare measures (Table SI3.6). All had an Acquisition, Transfer and Transition Policy in some form. Well-trained staff that are knowledgeable in species-specific chimpanzee behaviour patterns are essential for best practice, high welfare, PRT trainings, and accurate observations and daily monitoring, especially during the process of introductions and group formation (e.g., Fritz & Howell 2001; Laule & Whittaker 2001). The keepers from all zoos attended great ape related continuous professional development (CPD) workshops; including BIAZA conferences, ABWAK (Association of British and Irish Wild Animal Keepers) events and CPD training events run by GAWg. Most zoos provided funding to keepers for CPD for all participation, and funded some attendance.

**Table S2.6: Qualitative responses describing welfare measures used to assess chimpanzee welfare.**

| **Zoo** | **Welfare measures** |
| --- | --- |
| 1 | activity level, locomotion & posture, mobility, feeding, foraging, social behaviour, attitude to environment, physical condition, faeces/urine, animal-keeper interaction, visitor interaction, body score, abnormal behaviours, naturalistic behaviour, parental care, reproductive success |
| 5 | enclosure size, enclosure complexity, habitat imitation, privacy, escape routes, shelter, cleanliness, live plant use, protection from noise and vibration, enclosure condition, enclosure safety, separation facilities, temperature, filtration, lighting, enrichment frequency, enrichment success, enrichment variety, enrichment suitability, enrichment complexity, social group formation, breeding monitoring/ control, enclosure capacity for group size, group age, group harmony, mental health, abnormal behaviours, activity level, choices, body condition, hair/skin condition, visible injuries, water access, faecal score, on-going health problems, medication, training choice, regular training, training aid in welfare application, pr training |
| 6 | enclosure capacity for group size, adequate shelter, visible barriers, escape routes, specie-specific enclosure furnishings, temperature, water quality/testing, lighting, cleanliness, no pooling of water in enclosure, low dust levels, suitable substrate, indoors never smells of ammonia, suitable bedding, access to clean water, no animal is underweight, no animal is overweight, diet sheet, adequate quantity of food, food quality is good, little evidence of pest species, good physical health, no chronic health problems, survive to old age, animal can be visually individually identified, faecal samples can be readily collected, animals can be treated if needed, oral treatment is possible if needed, can be separated if needed, feeding methods address group hierarchy, all are well integrated, no stereotypic behaviours, other abnormal behaviours, enrichment |

**Health care management**  Veterinary or health care is essential for the best practice management of captive chimpanzees. Health issues highlighted in chimpanzees include cardiopulmonary problems, which are a major contributor to deaths in the captive population (Ross et al, 2022). Health monitoring is therefore a key component of good husbandry and high welfare, so long as it does not cause undue stress or suffering.

Four zoos had a formal Health Care Plan, but these ranged in detail from one that contained analysis of faecal samples and vaccinations to two that incorporated seven different procedures (Table SI3.7). Of the three zoos that had no Health Care Plan, two specified that keepers were responsible for performing daily checks. Zoos were asked to indicate the frequency with which a list of health measures were tested (daily, weekly, monthly, >6 monthly, >yearly, rarely, as required and never; Table S4 in SI). Although five zoos tested their chimps for cardiovascular health when required, we found that the participation in the International Primate Heart Project (Cardiff University, UK) and Ape Heart Project (Twycross Zoo, UK) to support a better understanding of cardiac disease in great apes was mixed across all facilities. Only one monitored parameters for the International Primate Heart Project on a monthly basis. Non-invasive health checks were conducted much more frequently, particularly assessing body condition for injuries wounds etc (carried out daily at all zoos except one, who responded as required), and activity levels (conducted daily at four zoos, weekly at one but as required at two).

**SI3.7. Qualitative responses regarding procedures included in Health Care Plans**

| **Zoo** |  |
| --- | --- |
| **2** | health care plan as formulated by IZVG; summary of document relevant to great apes; suspected disease reported immediately to IZVG; details on method and frequency of faecal collection and screening; details on post mortem arrangements and circumstances under which they are undertaken; testing required prior to the import and export of specimens; zoonotic disease surveillance and management; all this in addition to visual health checks conducted by keepers three times a day. |
| **3** | quarterly faecal sample testing for parasitology and bacteriology, pre-entry screening - TB interdermal test, haematology and biochemistry, chest x-rays, viral disease screen (siv, herpes, hepatitis), microbiology screen (salmonella, shigella, campylobacter, yersinia), physical examination |
| **4** | regular faecal samples provided to the on-site vet team |
| **6** | annual faecal screening, annual flu vaccine |

**S2.8: Responses regarding which health parameters are monitored/ tested.**

| **Zoo** | **1** | **2** | **3** | **4** | **5** | **6** | **8** |
| --- | --- | --- | --- | --- | --- | --- | --- |
| **cardiovascular health** |  |  |  |  |  |  |  |
| blood pressure | R | N | AR | AR | AR | AR | AR |
| echocardiogram | R | N | AR | AR | AR | AR | AR |
| electrocardiogram | R | N | AR | AR | AR | AR | AR |
| intl. Primate Heart Project | R | AR | AR | M | N | AR | AR |
| Great Ape Heart project | R | N | AR | N | N | AR | N |
| **Physical examinations** |  |  |  |  |  |  |  |
| ophthalmic | R | AR | AR | AR | AR | AR | AR |
| otic | R | AR | AR | AR | AR | AR | AR |
| dental | R | AR | AR | AR | AR | AR | AR |
| blood (parameters) | R | AR | AR | AR | AR | AR | AR |
| lymphatic | R | AR | AR | AR | AR | AR | AR |
| cardiovascular | R | AR | AR | AR | AR | AR | AR |
| respiratory | R | AR | AR | AR | AR | AR | AR |
| abdominal palpation | R | AR | AR | AR | AR | AR | AR |
| musculoskeletal | R | AR | AR | AR | AR | AR | AR |
| urogenital | R | AR | AR | AR | AR | AR | AR |
| neurologic | R | N | AR | AR | AR | AR | AR |
| **body condition (e.g. injuries, wounds, hair loss)** | AR | D | D | D | D | D | D |
| **Body Condition Score** | AR | N | R | N | N | N | AR |
| **weight** | >6M | AR | M | M | M | R | AR |
| **faecal sample (pathogenic bacteria/ parasites)** | >6M | R | >6M | >6M | >Y | >Y | >6M |
| **deworming** | >6M | N | AR | AR | >6M | AR | >6M |
| **urine (e.g. neopterin levels to monitor immune status)** | AR | AR | AR | AR | AR | AR | AR |
| **activity levels** | W | D | D | D | D | AR | AR |
| **vaccination** | N | N | AR | N | N | >Y | N |
| **joint/ muscular degeneration** | AR | D | AR | AR | AR | AR | R |
| **needs of geriatric individuals** | AR | D | D | AR | >Y | AR | AR |
| **needs of those with chronic conditions** | AR | D | AR | AR | >6M | AR | AR |

R: rarely; N: never, AR: as required, D: daily, M: monthly, >6M: up to every 6 months; >Y: up to yearly.

**REFERENCES**

Adang, O.M.J., Wensing, J.A.B., van Hooff, J.A.R.A.M. (1987). The Arnhem Zoo colony of chimpanzees Pan troglodytes: development and management techniques. International Zoo Yearbook. 26: 236-248.

Amsler, S.J. (2009). Ranging behavior and territoriality in chimpanzees at Ngogo Kibale National Park, Uganda. PhD Thesis. University of Michigan.

Angus, S. (1971). Water-contact behavior of chimpanzees. Folia Primatologica. 14: 51-58.

Baker, K.C. (1997). Straw and forage material ameliorate abnormal behaviors in adult chimpanzees. Zoo Biology. 16: 225-236.

Ban, S., Boesch, C. & Janmaat, K. L. (2014). Taï chimpanzees anticipate revisiting high-valued fruit trees from further distances. Animal Cognition. 17: 1353-1364.

Ban, S.D., Normand, E. (2019). Spatial cognitive abilities of foraging chimpanzees. In: The Chimpanzees of the Taï Forest. 40 years of research. In: C. Crockford, L. Vigilant, T. Deschner, & F. Leendertz (Authors) & C. Boesch & R. Wittig (Eds.). Cambridge: Cambridge University Press. pp. 440-450

Bates LA, Byrne RW. (2009). Sex differences in the movement patterns of free‐ranging chimpanzees (*Pan trgolodytes schweinfurthii*): foraging and border checking. Behavioral Ecology and Sociobiology. 64 (2): 247– 255.

Bayne, K. (1989). Environmental enrichment alternatives for laboratory nonhuman primates. In: Animal care and use in biomedical research regulations, issues and applications. (Ed.) Driscoll, J. Washington, DC: Animal Welfare Information Center. pp. 91-102.

Bloomsmith, M.A., Lambeth, S.P., Alford, P.L. (1991). The relationship between social behavior and captive female chimpanzees: Implications for managing chimpanzee (Pan troglodytes) groups. International Journal of Comparative Psychology. 4: 171-184.

Bloomsmith, M.A., Ross, S.K., Buchanan-Smith, K.C. (2000). Control over computer‐assisted enrichment for socially housed chimpanzees. American Journal of Primatology. 51: 45.

Bloomstrand et al. (1986). An objective evaluation of a behavior enrichment device for captive chimpanzees (*Pan troglodytes*). Zoo Biology. 5: 293-300.

Boesch, C., Boesch, H. (1984). Mental map in wild chimpanzees: An analysis of hammer transports for nut cracking. Primates. 25: 160-170.

Boesch, C., Boesch-Achermann, H. (2000). The chimpanzees of the Tai Forest: behavioural ecology and evolution. Oxford: Oxford University Press.

Brooks, J., Yoshimura, H., Taki, Y. (2021). Knowledge-based enrichment: Development of a novel enrichment device for captive chimpanzees. Zoo Biology. doi: 10.1002/zoo.21617.

Brosnan, S. F. & Hopper, L. M. (2013). Cooperation, behavioral diversity and inequity responses. In: Banaji, M. & Gelman, S. (Eds.). Navigating the Social World: What Infants, Children, and Other Species Can Teach Us. New York: Oxford University Press. pp 371-376.

Carlstead, K., Mench, J.A., Meehan, C., Brown, J.L. (2013). An epidemiological approach to welfare research in zoos: The Elephant Welfare Project. J. Appl. Animal Welfare Science. 4 (16): 319-337.

Chamove, A. S. (1989). Enrichment in chimpanzees: Unpredictable ropes and tools. RATEL (Journal of the Association of British Wild Animal Keepers) 16: 139.

Clark, F.E., Smith, L.J. (2013). Effect of a cognitive challenge device containing food and non-food rewards on chimpanzee well-being. American Journal of Primatology. 75: 807–816.

Clegg, I.L.K., Borger-Turner, J.L., Eskelinen, H.C. (2015). C-Well: The development of a welfare assessment index for captive bottlenose dolphins (*Tursiops truncatus*). Animal Welfare. 24: 267-282.

Clutton-Brock, T.H., Gillet, J.B. (1979). A survey of forest composition in the Gombe National Park, Tanzania. African Journal of Ecology. 17: 131-158.

Farmer, K.H. (2002). The behaviour and adaptation of reintroduced chimpanzees (*Pan troglodytes troglodytes*) in the Republic of Congo. PhD thesis, University of Stirling.

Fritz, J., Howell, S. (2001). Captive chimpanzee social group formation. In: Brent L, editor. The care and management of captive chimpanzees. San Antonio, TX: American Society of Primatologists. pp. 173–204

Holick, M.F. (1994). McCollum Award Lecture, Vitamin D: new horizons for the 21st century. The American Journal of Clinical Nutrition. 60: 619-630.

Kojima, S. (1990). Comparison of auditory functions in the chimpanzee and human. Folia Primatologica. 55: 62-72.

Lambeth, S.P., Bloomsmith, M.A. (1994). A grass foraging device for captive chimpanzees (*Pan troglodytes*). Animal Welfare. 3: 13-24.

Lambeth, S.P., Hau, J., Perlman, J.E., Martino, M., Schapiro, S.J. (2006). Positive reinforcement training affects hematologic and serum chemistry values in captive chimpanzees (*Pan troglodytes*). American Journal of Primatology. 68(3): 245-256.

Laule, G., Whittaker, M. (2001). The use of positive reinforcement techniques with chimpanzees for enhanced care and welfare. In: Special Topics in Primatology Volume 2 - The Care and Management of Captive Chimpanzees. Brent, L. (ed.). The American Society of Primatologists, San Antonio, TX. pp. 242-266.

Laule, G., Whittaker, M. (2007). Enhancing nonhuman primate care and welfare through the use of positive reinforcement training. Journal of Applied Animal Welfare Science. 10 (1): 31-38.

Maiki, S., Alford, P.L., Bloomsmith, M.A., Franklin, J. (1989). Food puzzle device stimulating termite fishing for captive chimpanzees (*Pan troglodytes*). *American Journal of Primatology.* (1 Suppl.). 71-78.

McCormick, W.D., Spink, A., Grieco, F., Krips, O., Loijens, L., Noldus, L., Zimmerman, P. (2012). Recognising & assessing positive welfare: developing positive indicators for use in welfare assessment. In: Measuring Behavior. Noldus Information Technology. pp. 241-243.

McDonald, S. (1994). The Detroit Zoo chimpanzees: exhibit design, group composition and the process of group formation. International Zoo Yearbook. 33: 235-247.

Meehan, C.L., Mench, J.A. (2007). The challenge of challenge: Can problem solving opportunities enhance animal welfare? Applied Animal Behaviour Science. 102: 246-261.

Normand, E., Boesch, C. (2009). Sophisticated Euclidean maps in forest chimpanzees. Animal Behaviour. 77: 1195-1201.

Paquette, D., Prescott, J. (1988). Use of novel objects to enhance environments of captive chimpanzees. Zoo Biology. 7: 15-23.

Perlman, J.E., Lambeth, S.P. Horner, V. et al. (2010). Videotaped demonstrator improves efficiency of training chimpanzees to urinate on cue. American Journal of Primatology. 72 (S1). 52.

Pomerantz, O., Terkel, J. (2009). Effects of positive reinforcement training techniques on the psychological welfare of zoo-housed chimpanzees (*Pan troglodytes*). American Journal of Primatology. 71: 687-695.

Quadros, S., Goulart, V.D.L., Passos, L., Vecci, M.A.M., Young, R.J. (2014). Zoo visitor effect on mammal behaviour: Does noise matter? Applied Animal Behaviour Science. 156: 78-84.

Reinhardt, V., Liss, C., Stevens, C. (1995). Restraint methods of laboratory nonhuman primates: a critical review. Animal Welfare. 4: 221-38.

Ross, S. R., Joshi, P. B., Terio, K. A., & Gamble, K. C. (2022). A 25-Year Retrospective Review of Mortality in Chimpanzees (Pan troglodytes) in Accredited U.S. Zoos from a Management and Welfare Perspective. Animals: 12(15). https://doi.org/10.3390/ani12151878

Sambrook, T.D., Buchanan-Smith, H.M. (1997). Control and complexity in novel object enrichment. Animal Welfare. 6: 207-216.

Shefferly, N., Fritz, J., Howell, S. (1993) Toys as environmental enrichment for captive juvenile chimpanzees (*Pan troglodytes*). Laboratory Primate Newsletter. 32 (2):7-9.

Sherwen, S.L., Hemsworth, L.M., Beausoleil, N.J., Embury A., Mellor, D.J. (2018). An animal welfare risk assessment process for zoos. *Animals*. 8: 130.

Shettleworth, S. J. (2010). Cognition, evolution and behavior, 2nd ed. Oxford, UK: Oxford University Press.

Strong, V., Moittie, S., Sheppard, M., White, K., Redrobe, S., Cobb, M., & Baiker, K. (2019). Idiopathic myocardial fibrosis in captive chimpanzees (*Pan troglodytes*). *Veterinary Pathology*, *57*, 183–191.

Videan, E.N., Fritz, J., Schwandt, M.L., Smith, H.F., Howell, S. (2005). Controllability in environmental enrichment for captive chimpanzees (*Pan troglodytes*). Journal of Applied Animal Welfare Science. 8(2): 117-130.

Wallace, G. (1988). Improving life for primates. Caring for Animals. 5(1): 3.

Wessling, E.G., Deschner, T., Mundry, R., Pruetz, J.D., Wittig, R.M., Kuehl, H.S. (2018). Seasonal variation in physiology challenges the notion of chimpanzees (*Pan troglodytes verus*) as a forest-adapted species. Frontiers in Ecology and Evolution. 6: 60.

Whiten, A. (2011). The scope of culture in chimpanzees, humans and ancestral apes. Phil. Trans. R. Soc. B. 366 (1567): 997-1007.

Whitham, J.C., Wielebnowski, N. (2009). Animal-based welfare monitoring: Using keeper ratings as an assessment tool. Zoo Biology. 28: 545-560.

Wood, W. (1998) Interactions among environmental enrichment, viewing crowds and zoo chimpanzees (*Pan troglodytes*). Zoo Biology. 17: 211-230.

Zaragoza, F., Ibáñez, M., Mas, B., Laiglesia, S., Anzola, B. (2011). Influences of environmental enrichment in captive chimpanzees (*Pan troglodytes ssp.)* and gorillas (*Gorilla gorilla gorilla*): behavior and faecal cortisol levels. Revista Cientifica. 5: 447-456.
